# Real-World Evidence Evaluating Teclistamab in Patients with Relapsed/Refractory Multiple Myeloma: A Systematic Literature Review

**DOI:** 10.3390/cancers17071235

**Published:** 2025-04-05

**Authors:** Benjamin Derman, Carlyn Tan, Ian Steinfield, Florence R. Wilson, Dee Lin, Bingcao Wu, Mariana Fernandez, Jessica Fowler, Agne Paner-Straseviciute, Nina Kim, Margaret Doyle, Alexander Marshall, Jessica Cheadle, Sam Keeping, Jane Jijun Liu

**Affiliations:** 1 Section of Hematology/Oncology, University of Chicago Medical Center, Chicago, IL 60637, USA; 2 Division of Hematology/Oncology, Memorial Sloan Kettering Cancer Center, New York, NY 10065, USA; tanc4@mskcc.org; 3 Precision AQ, Boston, MA 02110, USA; ian.steinfield@precisionaq.com; 4 Precision AQ, Vancouver, BC V6J 1H2, Canada; florence.wilson@precisionaq.com (F.R.W.); sam.keeping@precisionaq.com (S.K.); 5 Janssen Scientific Affairs, LLC, Horsham, PA 19044, USA; dlin@its.jnj.com (D.L.); bwu34@its.jnj.com (B.W.); jfowler4@its.jnj.com (J.F.); apanerst@its.jnj.com (A.P.-S.); nkim32@its.jnj.com (N.K.); jessicacheadle@hotmail.com (J.C.); 6 Janssen-Cilag S.A., 28703 Madrid, Spain; mfern107@its.jnj.com; 7 Janssen Sciences Ireland, D24WR89 Dublin, Ireland; mdoyle11@its.jnj.com; 8 Janssen Global Services, LLC, Raritan, NJ 08869, USA; amarsh16@its.jnj.com; 9 Illinois CancerCare, Peoria, IL 61615, USA; jliu@illinoiscancercare.com

**Keywords:** systematic literature review, real-world evidence, relapsed–refractory multiple myeloma, bispecific antibodies, B-cell maturation antigen, teclistamab, cytokine release syndrome, immune effector cell-associated neurotoxicity syndrome

## Abstract

**Background**: Teclistamab (TEC) is the first B-cell maturation antigen-directed bispecific antibody approved in 2022 by the European Medicines Agency and Food and Drug Administration for triple-class exposed relapsed/refractory multiple myeloma (RRMM). **Objectives**: As TEC is increasingly used in real-world (RW) settings, this study seeks to gather existing RW evidence on effectiveness, safety, healthcare resource utilization, and clinical practices associated with TEC. **Methods**: A systematic literature review was performed to identify RW observational studies of TEC-treated adults with RRMM from 2023 to June 2024. **Results**: Sixty-one records representing 41 unique studies were included; sample sizes ranged from 8 to 572 patients. Where reported, median follow-up ranged from 2.3 to 33.6 months, and >65% of the patients would have been ineligible for the pivotal trial of TEC (MajesTEC-1) in all but one study. In eight studies with ≥50 patients and ≥3 months follow-up, overall response rates were 59–66% and cytokine release syndrome (CRS) rates were 18–64%. Tocilizumab use for CRS management was reported in 14 studies, with two indicating CRS rates of 13% and 26% when used prophylactically. Survival and infection outcomes showed wide variability due to short follow-up in most studies. **Conclusions**: Overall, early RW effectiveness and safety outcomes of TEC were comparable to findings from MajesTEC-1.

## Simple Summary

Teclistamab belongs to a class of drugs known as bispecific antibodies. It is used to treat patients with relapsed or refractory multiple myeloma who have tried several other types of therapy. Teclistamab was initially studied in the MajesTEC-1 clinical trial and has been increasingly used in clinical practice since its approval in October 2022. In this study, we perform a systematic literature review to gather evidence on effectiveness, safety, healthcare resource utilization, and prescribing patterns associated with teclistamab in real-world observational studies. Results indicate that patients treated with teclistamab across health centers in the real-world have similar outcomes to patients treated in the clinical trial setting. As patients with relapsed or refractory multiple myeloma have a high disease burden and limited treatment options, results from this study will inform healthcare providers and health policy makers of the therapeutic benefits and safety profile of teclistamab in this patient population.

## 1. Introduction

Multiple myeloma (MM), a plasma cell malignancy, is the second most common hematologic cancer in the United States (US), with an estimated 35,780 new cases (1.8% of all cancer diagnoses) and 12,540 deaths (2.0% of all cancer deaths) in 2024 [1]. While 5-year survival rates of MM have more than doubled since the 1970s to a rate of 61.1% [1,2], the disease is typically characterized by a series of responses and relapses meaning patients ultimately require multiple lines of therapy [3]. It imposes a substantial healthcare burden, as patients progress through successive lines of therapy; a recent retrospective database study found that all-cause healthcare costs averaged USD 35,760 per patient per month among patients with four or more prior lines of therapy [4]. After the three main classes of drugs for MM (proteasome inhibitors [PI], immunomodulatory imide drugs [IMiD], and anti-CD38 monoclonal antibodies [CD38 mAb]) have failed patients, few effective options remain for relapsed/refractory multiple myeloma (RRMM). Deciding on the next steps in treatment requires clinicians and patients to consider the pace of disease progression, patient comorbid conditions, potential clinically relevant toxicities, and the timing as well as financial implications of treatment [5].

The treatment landscape for RRMM has evolved considerably with the introduction of innovative targeted therapies, including B-cell maturation antigen (BCMA)-targeted chimeric antigen receptor (CAR) T-cell and bispecific therapies since 2020 [6]. Teclistamab-cqyv (TECVAYLI^®^, Janssen Biotech, Horsham, PA, USA) is the first-in-class BCMA-targeted bispecific antibody (BsAb) approved by the European Medicines Agency (EMA) and the Food and Drug Administration (FDA) in August and October 2022, respectively, for adult patients with RRMM who have previously received at least three prior lines of therapy in Europe or at least four prior lines of therapy in the US, including a PI, IMiD, and CD38 mAb.

Teclistamab (TEC) is administered subcutaneously, initially using a step-up dosing (SUD) schedule to lower the risk of cytokine release syndrome (CRS) and neurotoxicity, followed by weight-based weekly or every-other-week treatment doses; patients are recommended to be hospitalized for 48 h after SUD for monitoring. Although TEC SUD is recommended to be delivered in an inpatient setting, since its launch, clinicians have been exploring different care models, including outpatient administrations, with the goal of reducing healthcare resource utilization (HCRU) and improving treatment experiences while ensuring patient safety [7,8,9].

MajesTEC-1, the initial phase I/II trial reporting on the efficacy and safety of TEC, demonstrated an overall response rate (ORR) of 63% [10,11], including 46% of patients achieving complete response (CR) or better [11]. CRS occurred in 72% of patients (most CRS events were grade 1 or 2 in severity while one patient [0.6%] experienced a grade 3 event in the setting of an ongoing infection), and 3% developed immune effector cell-associated neurotoxicity syndrome (ICANS) [10]. MajesTEC-1 utilized stringent eligibility criteria that excluded patients with significant disease burden, such as those with organ dysfunction, poor performance status, serious comorbid conditions, severe cytopenia, and/or prior BCMA-directed therapy exposure; however, these characteristics are common in real-world (RW) practice [10,12]. As a result, patients treated in MajesTEC-1 may not have been representative of all indicated patients in a RW setting [12]. Several consortia and institutions have disseminated reports on early RW outcomes and safety with TEC. As TEC is increasingly being used in RW settings with a growing amount of RW evidence being published, this systematic literature review (SLR) aims to identify and summarize the latest RW outcomes of TEC, including effectiveness, safety, healthcare provider practices, and associated HCRU.

## 2. Materials and Methods

This SLR was conducted according to the Preferred Reporting Items for Systematic Reviews and Meta-Analyses (PRISMA) guidelines [13]. This review has not been registered. The full study protocol and checklist are provided in Table A1 and the Supplementary Materials for reference.

Study eligibility criteria were defined in terms of the population, intervention, comparator, outcome, and study design (PICOS) structure outlined in Table A1 which guided the identification and selection of studies for the SLR. The target population included adult patients (≥18 years) with MM. The only intervention of interest was TEC, but no restrictions were applied to comparators. No restrictions were applied to outcomes to ensure that all relevant evidence was captured. Observational RW studies (prospective and retrospective) were included, while clinical trials, pooled analyses of clinical trials, case reports, case series, and narrative reviews were excluded. Publications of SLRs were excluded, but the bibliographies of relevant SLRs were screened for any relevant citations not otherwise captured in the primary searches (i.e., these references served only as secondary sources to ensure that all studies meeting the eligibility criteria were identified). Only English-language publications of full-text articles and conference materials (i.e., abstracts, posters, or oral presentations) were included, and a time restriction from 2023 to 2024 was applied.

Relevant studies were identified by searching the following databases through the Ovid platform: Medical Literature Analysis and Retrieval System Online (MEDLINE) and Excerpta Medica database (Embase). The specific search algorithms included a combination of indexing and free-text terms (see search terms in Table A2 and Table A3). The population terms were adapted from existing reviews [14,15], and terms for the generic and brand names of TEC were incorporated.

The main database searches were augmented with searches of 15 specific clinical or managed care conference proceedings from 2023 and 2024 (Table A5). The Northern Light database was used to search for studies from conferences that were indexed in the database (see search terms in Table A4), while conference websites were hand searched for the remaining conferences. Database searches were conducted at the end of May 2024, and further hand searches were conducted through the end of June 2024 to ensure all relevant materials were captured through the first half of 2024.

One reviewer performed all citation screening and data extraction, with quality checks performed by a second reviewer; a third reviewer was included to reach a consensus on any remaining discrepancies, where necessary. Screening decisions and extracted data were stored and managed in Microsoft Excel (Microsoft, Redmond, WA, USA). The study identification and selection process was summarized with a PRISMA flow diagram [13]. Kaplan–Meier (KM) curves for progression-free survival (PFS) and overall survival (OS) were extracted using DigitizeIt (DigitizeIt, Braunschweig, Germany) and reconstructed using an algorithm published by Guyot et al., 2012 [16]. The Newcastle–Ottawa Scale (Ottawa Hospital Research Institute, Ottawa, ON, Canada) was used to assess the quality of observational studies where full-text publications were available (Table A6). The scale utilizes a ‘star system’ to judge (i) the selection of the study groups, (ii) the comparability of the groups, and (iii) the ascertainment of either the exposure or outcome of interest for case–control or cohort studies, respectively.

## 3. Results

### 3.1. Study Characteristics

**Figure 1:**
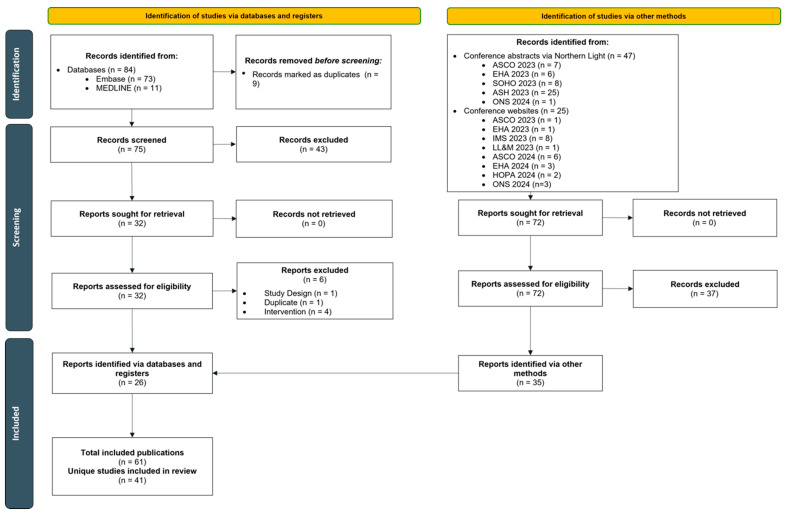
PRISMA flow diagram of included studies. Abbreviations: ASCO, American Society of Clinical Oncology; ASH, American Society for Hematology; EHA, European Hematology Association; HOPA, Hematology/Oncology Pharmacy Association; IMS, International Myeloma Society; LL&M, Lymphoma, Leukemia & Myeloma Congress; ONS, Oncology Nursing Society; SOHO, Society of Hematologic Oncology.

Of the 156 records identified across all sources, a total of 61 publications (five full-texts [12,17,18,19,20] and 56 conference abstracts, posters, or oral presentations) representing 41 unique studies evaluating TEC in a RW setting were identified (Figure 1). The included studies spanned seven countries in North America and Europe. Thirty-five of the 41 studies were retrospective in design. Fifteen studies (36.6%) reported results pooled from multiple centers, while 25 studies (61.0%) reported experience from a single center. Most studies (n = 34) were chart reviews, and seven reported data gathered from secondary databases. In 40 studies, sample sizes ranged from 8 to 572 patients. One study using the FDA Adverse Event Reporting System database reported 719 adverse event (AE) cases of TEC, rather than the event rates among patients [21], and, therefore, was not included in the results summary. Median follow-up (mFU) duration was reported in 19 studies (46.3%) and ranged from 2.3 [22] to 33.6 months [23]. This current review focused on eight studies with sample sizes ≥50 (range: 52 to 419) and mFU ≥3 (range: 3.1 to 9.5) months, as studies with larger sample sizes may be more generalizable to the wider population and those with longer follow-up duration may help to accurately evaluate AEs and outcomes (the median time to CR or better was 4.6 months [24] in MajesTEC-1). Exceptions were made for seven studies in order to report on data from novel topics including prophylactic tocilizumab (TCZ) use, less frequent dosing, and step-up dosing in the outpatient setting that have not been extensively evaluated or reported in the real-world yet and where data are primarily limited to smaller sample sizes with shorter follow-up periods. Studies not reporting mFU were not included in this synthesis due to the limited interpretability of the outcomes.

Study characteristics of studies with ≥50 patients and ≥3 months mFU are presented in Table 1 (Table A7 summarizes all 41 included studies). ORR and CRS rates were the most frequently reported effectiveness and safety measures, respectively. AE management using TCZ, length of hospital stay during TEC SUD, ICANS rates, and infections were also frequently reported. Common subpopulations included patients with prior anti-BCMA exposure and those receiving outpatient SUD. Studies that included other interventions alongside TEC were included when TEC-only subpopulation data were available (labeled as “TEC treatment group” in subsequent tables). Based on the Newcastle–Ottawa scale, the five included full-text publications [12,17,18,19,20] were high quality regarding study population selection and ascertainment of outcomes. However, all five studies had a non-comparative design and could not be compared directly.

### 3.2. Population Characteristics

Population characteristics of studies with ≥50 patients and ≥3 months mFU are reported in Table 2 and Table 3. Summaries of all included studies are available in Table A8 and Table A9, with additional characteristics provided in Table A12 and Table A13. Within the overall populations, the median age ranged from 65 [35] to 71 [40] years, and 63.4% [35] to 76.0% [40] of the patients were White. High-risk cytogenetic abnormalities were reported in six studies and were present in 33% [17] to 71% [40] of the patients. Five studies consistently defined high-risk cytogenetics as having one of the following abnormalities: del17p, t(4;14), or t(14;16) [12,19,20,37,40]; four of these studies included 1q gain/amp [12,19,37,40]; two studies included t(14;20) [37,40]; and one study included monosomy 17 [37]. The remaining study did not provide a definition [17].

Extramedullary disease (EMD) was present in 19% [37] to 44% [19] of the patients in six chart review studies, where documented. One study utilizing data from a multi-center MM electronic medical record (EMR) database reported that EMD diagnosis codes were present in 5.7% of the patients [25]. Of these studies, two defined EMD as any plasmacytomas [17,25] and four defined EMD as plasmacytomas not associated with the bone [12,19,37,40]. Only one study did not provide a definition for EMD [20]. The median number of prior lines of therapy ranged from five [35] to seven [17], such therapies including patients with prior BCMA-directed therapy exposure. Other prior lines of therapy for all included studies can be found in Table A12.

MajesTEC-1 ineligibility was reported in three studies: ≥70% of patients were ineligible in two studies [12,37] and one study reported 39% of the patients as ineligible [20]. The most common reasons for ineligibility in these studies included prior BCMA-directed therapy (37% [20] to 53% [12]), a poor performance status (Eastern Cooperative Oncology Group [ECOG] ≥2; 33% [12]), cytopenia(s) (31% [12]), and/or renal impairment/failure (12% [37] to 13% [12]). Additionally, individual cytopenia ranges across studies with ≥50 patients and ≥3 months mFU included anemia (25% [12] to 51% [25]), neutropenia (2% [12] to 22% [25]), and thrombocytopenia (20% [12]).

### 3.3. Outcomes

The following sections summarize the most frequently reported outcomes of interest for the overall populations in the included studies with ≥50 patients and ≥3 months mFU, focusing on the latest timepoint in each study. Table A10 and Table A11 provide outcome summaries for all included studies.

#### 3.3.1. Effectiveness Outcomes

Six of the eight studies with ≥50 patients and ≥3 months mFU reported on effectiveness outcomes (Table 4), and variability in response and survival rates was observed across these studies. Among these studies, ORR (partial response [PR] or better) ranged from 59% [20] to 66% [12] (n = 6 studies), very good partial response (VGPR) or better ranged from 38% [17] to 51% [19] (n = 6 studies), and CR or better ranged from 19% [37] to 29% [12] (n = 4 studies).

Where reported, PFS and OS varied across studies with ≥50 patients and ≥3 months mFU, as shown in Table 4. Among the studies reporting the median PFS (n = 5 studies; mFU 3.1 [17] to 5.5 [20] months), the median PFS ranged from 5.4 [12] to 13 [37] months and was not reached in two studies [17,19]. The 6-month PFS rate was similar across studies, where reported (52% [19] to 58% [37] in three studies; mFU 3.5 [19] to 9.5 [40] months). Among the studies reporting OS, the median OS was not reached in three studies (mFU 3.5 [19] to 5.5 [20] months), while one reported a median of 15 months (mFU 5 months [37]). The 6-month OS rate ranged from 70% [12] to 80% [19] (n = 3 studies; mFU 3.5 [19] to 5 [37] months).

#### 3.3.2. Safety Outcomes

Safety outcomes were reported in six of the eight studies (Table 5), with variability in the proportion of patients experiencing AEs observed across all studies. All six studies reported CRS rates, while three studies reported ICANS rates and four studies reported infection rates in the overall population. Among these studies, the proportion of patients who developed any grade of CRS ranged from 18% [25] to 64% [12] (n = 6 studies), and a small proportion of patients (0.5% [37] to 4.5% [19]) experienced grade ≥3 CRS (n = 5 studies). When stratified by data source and care models, any grade CRS rates from chart reviews of patients with inpatient monitoring ranged from 52% [17] to 64% [12], one study reported a CRS rate of 36% [37] from chart reviews of patients with outpatient monitoring, and one study reported a CRS rate of 18.4% [25] from secondary databases (e.g., payer claims, EMRs), as identified by the International Classification of Diseases 10th Revision codes. Three studies reported any grade ICANS rates ranging from 4% [25] to 14% [12], and three studies reported grade ≥3 ICANS rates ranging from 0% [25] to 4.5% [19]. Any grade infections were experienced by 31% [12] to 60% [37] of the patients (n = 4 studies), and approximately 26% of the patients had grade ≥3 infections (n = 2 studies) [19,20]. Two studies reported the impact on CRS rates when using prophylactic TCZ [42,43], as described in more detail below (Section 3.3.3).

#### 3.3.3. Healthcare Provider Practices and Resource Utilization

The most frequently reported outcome related to healthcare provider practices was TCZ use in CRS management, which was reported in 12 studies. Among the five studies with ≥50 patients and ≥3 months mFU, TCZ usage ranged from 15% [25] to 41% [12] in the overall population. Additionally, two studies with any sample size and any mFU reported on prophylactic TCZ use in the inpatient setting. Kowalski (2023) only included patients (n = 31) treated with prophylactic TCZ before the TEC treatment, and there was a CRS rate of 13% (all grade 1; 95% CI 4, 30; *p* < 0.01) [42]. Marin (2023) assessed patients treated with prophylactic TCZ prior to the second TEC SUD (n = 38) and compared them to a cohort of patients who received TEC without the prophylactic TCZ (n = 15) [43]. The cohort with the prophylactic TCZ had a 26% rate of any grade CRS, while the cohort without the prophylactic TCZ had a much higher rate of any grade CRS, at 73% [43]. Among the patients in the prophylactic TCZ cohort who experienced CRS events (n = 10), the majority of the events were grade 1 (n = 8), while only two patients had grade 2 or 3 events; CRS grades for the cohort without the prophylactic TCZ were not reported [43].

The most frequently reported HCRU outcome was hospital length of stay (LOS) during TEC SUD for inpatient TEC administration or AE management if hospitalized after an outpatient SUD administration. Three studies with ≥50 patients and ≥3 months mFU reported an inpatient SUD administration model, with the median LOS ranging from 8 days [37] to 9 days [12,34]. Two studies with any sample size and any mFU reported LOS data for 1-3-5 and 1-4-7 SUD schedules. Kawasaki (2024) reported a mean LOS of 7.6 days for the 1-3-5 schedule and 9.2 days for the 1-4-7 schedule. Graf (2024) reported a median LOS of 6 days for the 1-3-5 schedule and 9 days for the 1-4-7 schedule. Two studies reported that LOS for inpatient SUD decreased over time: Banerjee (2023a), using nationally representative payer claims data, reported a mean LOS of 11.4 (SD 9.0) days for patients who initiated TEC within the first four months of FDA approval (through February 2023), and a mean LOS of 7.0 (SD 1.4) days for patients who initiated TEC in the most recent month (July 2023 by the data cut-off) [44]. Similarly, Banerjee (2023b), using a multi-center MM EMR database, reported a median LOS of 11 days in December 2022 and a median LOS of 6.5 days in May 2023 [25]. Additionally, three studies (any sample size and any mFU) with patients undergoing outpatient SUD reported LOS for hospital admissions at any time due to AEs, with medians of 1.5 days [45], 2 days [46], and 4 days [47] per admission, respectively.

Three recently published studies with ≥50 patients and ≥3 months mFU reported the proportion and timing of patients switching from weekly dosing to less frequent dosing. Tan (2024) reported that 30 patients (34.9%) at a single academic center switched from every week (QW) to every 2 weeks dosing (Q2W) and two patients (2.3%) switched from QW to every 4 weeks (Q4W), with a median time to switch of 3.3 months, for the primary reason of achieving ≥PR (n = 23) and/or safety (n = 14) [40]. Among these patients who switched the dosing frequency, the 6-month PFS rate post-switch was 90% after an mFU of 6.4 months after switching [40]. Two studies, using nationally representative secondary data, estimated the probability and timing of patients switching to less frequent dosing using KM analysis. The probability of switching to less frequent dosing (32 out of 39 switchers went from QW to Q2W [26]; 58 out of 78 switchers went from QW to Q2W [35]) after three months was 15.5% [26] and 19.0% [35], and this increased to 38.3% [26] and 38.6% [35], respectively, at six months. The median time to switch was 8.5 months in one study [26] and it was not reached at an mFU of 4.2 months in the other [35].

## 4. Discussion

This is the first SLR to consolidate RW studies of TEC, globally. Wide variance was observed in the effectiveness and safety of TEC across the entire evidence base, with ORR ranging from 44% to 87%, CRS rates ranging from 6% to 85% depending on data sources and care models, and ICANS being present in 0% to 23% of the patients. When restricting the review to larger studies that included ≥50 patients with an mFU of ≥3 months, the range of results narrowed: ORR ranged from 59% to 66%, CRS rates ranged from 18% to 64% depending on data sources and care models, and ICANS rates ranged from 4% to 14%. Wide variability in PFS and OS estimates and limited data on infection were observed given the short follow-up time in most studies, and this trend was still observed when only considering studies with larger sample sizes. Early RW use of TEC was characterized by administration to a diverse group of patients, including minorities, patients with significant comorbid conditions, high-risk features, and those with prior BCMA-directed therapy exposure.

The collation of these data provides early insights into how RW outcomes of TEC compare with the results of the MajesTEC-1 trial (Table A15), taking into account that most RW studies report that more than half of the patients would have been ineligible for the MajesTEC-1 trial. Patients in the RW differ from the trial populations, since they are not required to meet stringent eligibility criteria and may have more comorbidities and less hematopoietic or organ reserve [10,12]. Additionally, with TEC being a first-in-class BCMA BsAb and with these studies capturing data from the first year since TEC approval, the patients included in RW studies may have had advanced diseases and may have been more heavily pretreated. Despite this, the early effectiveness and safety profile of TEC in RW practice appeared comparable with MajesTEC-1.

The ORR in MajesTEC-1 was 63% [10]; studies evaluating ORR in this SLR also provided similar results, with RW evidence showing a range of 59% [20] to 66% [12] among the studies with ≥50 patients and ≥3 months mFU. Despite the similarities in ORR, the depth of response differed in the early RW patients. In MajesTEC-1, 46% of the patients achieved a response of CR or better [11], whereas the CR rates ranged from 19% [37] to 29% [12] in the early RW setting. In RW practice, this may be a reflection of the hard-to-treat population which included heavily pretreated patients, high-risk features, significant comorbidities, and/or patients with prior exposure to BCMA-directed therapy, as well as the inability of the short follow-up period of the studies evaluated to assess CR or better. In RW practice, it may not be possible to undergo a bone marrow biopsy as frequently to confirm International Myeloma Working Group CR status, a fact which may result in the misclassification of responses as VGPR and result in an underestimated rate of CR or better. Furthermore, the median time to CR or better in MajesTEC-1 was 4.6 months [24], but, in RW studies, the follow-up was short, with only eight studies having an mFU ≥3 months, with a range of 3.1 to 9.5 months. This short follow-up in the RW studies may have limited the ability to assess the deepening of responses due to the delayed clearance of paraprotein following tumor killing [48], and thus may be unable to measure the best response, PFS, or OS effectively. Moreover, clarifying the duration between the patient’s prior BCMA-directed therapy and the first TEC dose in patients with prior exposure to BCMA-directed therapy will also be paramount to interpret their survival outcomes.

The rate of any grade CRS in the MajesTEC-1 clinical trial was 72% [10], which was greater than the range reported in RW studies with ≥50 patients and ≥3 months mFU (18% [25] to 64% [12]). However, the CRS rates in RW studies that used prophylactic TCZ (13% and 26%) [42,43] were similar to the subgroup in the MajesTEC-1 cohort that received prophylactic TCZ (26%) [24]. There was notable variation in CRS rates across the included studies, possibly due to differences in data sources, data collection methods, and the care models for CRS prophylaxis and monitoring. Patients found within secondary databases (e.g., payer claims, EMR) may have lower rates due to their reliance on diagnostic codes; for example, healthcare professionals may not code fever as CRS or code fever at all, and diagnosis codes specifically for CRS may not always be used. Chart reviews of patients with inpatient monitoring may represent a more rigorous definition of RW CRS rates with TEC, i.e., data are abstracted directly from physician’s notes, and CRS-relevant signs and symptoms are continuously monitored and recorded systematically in the hospital setting. However, it is important to note that these definitions may differ between settings and may not always be used. Data on patients who received outpatient monitoring tend to have lower rates compared to inpatient monitoring, as they may have different triggers and thresholds of CRS reporting and, sometimes, may rely on patient-reported symptoms. These differences in data sources and settings differ from the MajesTEC-1 trial as well, and, therefore, should be considered while interpreting results.

Studies included in the SLR also did not report if general neurotoxicity and ICANS were assessed together or if the reported value was for ICANS alone. Including neurotoxicity with ICANS may explain the higher rate of ICANS in the RW setting (4% [25] to 14% [12] among the studies with ≥50 patients and ≥3 months mFU) versus that observed in MajesTEC-1 (3%), as neurotoxicity symptoms cover a wide spectrum and definitions vary across practices.

Infection rates were sparsely reported across the RW studies, limiting the ability to analyze these data; among the larger studies with ≥3 months mFU, only four studies reported any grade infections (31% [12] to 60% [37]) and only two studies reported grade ≥3 infections (26% [19] and 27% [20]). MajesTEC-1 reported a higher rate of infections (79%), with 55% of the patients experiencing grade 3 or 4 infections [11], though this was in the context of longer follow-ups. Unlike CRS and ICANS, both of which occur at the beginning of the treatment and are less dependent on follow-up times in RW studies, infection risk persists throughout the treatment; therefore, studies with shorter follow-up times may underreport infection rates. Additionally, the enrollment of patients for MajesTEC-1 occurred concurrently with the onset of the COVID-19 pandemic, before the widespread vaccine availability and use, a fact which may have resulted in higher infection rates among MajesTEC-1 patients compared to the RW infection rates reported in the studies considered here, all of which were conducted in the post-pandemic period. Guidelines agreed upon by expert consensus have since been published, allowing for optimal treatment of infections during TEC treatment [49,50]. With the adoption of infection management guideline recommendations for MM, other reasons for lower RW infection rates could be routine vaccination against COVID-19 and prophylactic intravenous immunoglobulin use for hypogammaglobulinemia and antibiotic use. Given the short follow-up durations, these results must be interpreted with caution, and data on long-term infection prophylaxis and management will need to be assessed in future work.

This review also provides initial insights into healthcare provider practices and HCRU associated with TEC in the RW setting. The use of TCZ during SUD and other medications often used for managing AEs associated with TEC were frequently reported in the studies included in the SLR. Although preliminary results showed the benefits of using prophylactic TCZ in Marin (2023) [43] and Kowalski (2023) [42], further studies with larger sample sizes are warranted. Based on studies published in 2023 and the first half of 2024, most patients received TEC SUD in an inpatient setting, with some institutions starting to implement outpatient SUD models [7,9,37,45,51]. This is reflected in a recent study that conducted a panel interview of clinicians from 20 practices across 13 states who were among the first ones to start TEC in their practices after FDA approval. Within seven months of approval, 74% of these practices provided SUD exclusively in an inpatient setting, while 26% provided SUD in an outpatient or hybrid setting; all participating practices using inpatient SUD expressed desire of moving to outpatient SUD for BsAb in the future [52]. In two secondary database analyses that monitored inpatient LOS for SUD over time, a decreasing trend was observed [25,44]. This observation might be due to improved familiarity with TEC among providers over time, availability of well-established AE management protocols, and quality improvement models that institutions might have implemented to reduce HCRU. Furthermore, two studies using large databases of medical records suggest that patients were able to complete SUD without delay, with the two-day and three-day dosing schedules being the most common, and that the majority of the patients were taking pre-medications as recommended [25,53].

Recent studies with RW data available for patients who switched to a less frequent dosing schedule (most frequently switching to Q2W) have shown results in line with the MajesTEC-1 trial. In MajesTEC-1, patients were allowed to switch from weekly TEC doses to every-other-week if they achieved a PR or better after four or more cycles in phase 1 or a CR or better for six or more months in phase 2 [10,11]. With over two years of follow-up, TEC demonstrated deep and durable responses and reduced new onset grade ≥3 infections over time, aligning with the median time to switch to Q2W dosing (~11 month mFU), including in patients who switched to less frequent dosing [11]. The reduction in new onset grade ≥3 infections may also be impacted by the increasing usage of IVIG and prophylaxis [11]. Three RW studies identified in the SLR reported on less frequent TEC dosing, mainly with a Q2W schedule, among patients who switched [26,35,40]; Q4W was rarely observed within these studies. Tan (2024) reported a 6-month PFS rate of 90% in patients who switched to less frequent dosing based on response or for safety management [40]; longer follow-ups are needed to understand the long-term impact on PFS and OS.

This SLR has some limitations. First, the recent approval of TEC resulted in the restriction of studies to the 2023 and 2024 calendar years, leading to an evidence base consisting of mostly conference materials with only five full texts. As conference abstracts may not include as detailed information as full-text publications, these results should be interpreted with caution. Due to the publication date restriction, most studies included patients who initiated TEC within 1 year of approval. This resulted in short follow-up periods and small sample sizes; longer follow-up time is needed for RW outcomes to mature. Many of these early patients were expected to be sicker and with a higher disease burden, contributing to the high percentage of reported MajesTEC-1 ineligibility. Despite this, the early effectiveness and safety outcomes remain consistent with the clinical trial. When sample sizes allow it, results may be stratified by MajesTEC-1 eligibility to provide a more balanced comparison with clinical trial results. Further, given the short follow-up periods, outcomes such as PFS, OS, and infection rate often need to be considered carefully, as current data may not reflect true estimates over time. Finally, numbers at risk over time or the total number of events are needed for reasonable accuracy during KM curve extraction and recreation [16], but this information was not available in several studies. As a result, comparisons made through the KM curves are limited and need to be interpreted cautiously. These limitations are further affected by a wide variation in the data due to differences in sample sizes, differences in data sources, and differences in RW clinical practices across health centers. Due to these limitations, a meta-analysis with patient-level data would be an appropriate next step once the data mature.

## 5. Conclusions

In conclusion, this SLR summarizes the initial RW evidence available for TEC. Variation and immature data limit the interpretation of long-term outcomes; however, results from studies with larger sample sizes and ≥3 months mFU suggest that the early effectiveness and safety profiles for TEC in the RW are comparable to those from the pivotal trial, even for patients who were sicker, with a high disease burden, and which would not have met the eligibility criteria for MajesTEC-1.

## Figures and Tables

**Table 1:** Summary of study characteristics and outcomes evaluated in studies with ≥50 patients and ≥3 months median follow-up.

Study ID	Study Design	Data Source	Chart Review vs. Secondary Database	Setting	Study Timeframe	mFU (Months)	Sample Size	Relevant Outcomes Evaluated
Banerjee 2023b [25,26]	Retrospective	Acentrus MM electronic medical records (EMRs)	Secondary database	Academic centers/community-based hospitals, multi-center	October 2022–November 2023	5.1	247	LOS, CRS, ICANS, step-up dosing schedule, time to next treatment (TTNT), or death
Dima 2023 [12,27,28,29,30]	Retrospective	Patients treated at USMIRC centers	Chart review	Academic, multi-center	August 2022–August 2023	3.8	106	CRS, ICANS, infections, ORR, DOR, OS, PFS, TCZ use in AE management, hospitalizations
Firestone 2023 [17,31,32,33]	Retrospective	Patients treated at the Memorial Sloan Kettering Cancer Center	Chart review	Academic, single-center	November 2022–July 2023	3.1	52	Survival, PFS, ORR, safety
Mohan 2024 [19,34]	Retrospective	Patients treated at five US academic centers	Chart review	Academic, multi-center	NR	3.5	110	CRS, ICANS, infections, ORR, best response, LOS, IVIG use in AE management, TCZ use in AE management
Pianko 2024 [35]	Retrospective	Komodo Healthcare MapTM	Secondary database	Multi-center	October 2022–December 2023	4.2	419	Dosing schedule of teclistamab, time to less frequent dosing, TTNT
Riedhammer 2024 [20,36]	Retrospective	Patients treated at 18 German centers	Chart review	Multi-center	July 2022–October 2023	5.5	123	Time to response, best response, ORR, PFS, infections
Tan-Asoori 2023 [37,38,39]	Retrospective	Patients treated at IMF-associated centers	Chart review	Academic, multi-center	NR—October 2023	5	204	ORR, OS, PFS, CRS, ICANS, TCZ use in AE management, IVIG use in AE management, LOS
Tan 2024 [40,41]	Retrospective	Patients treated at the Memorial Sloan Kettering Cancer Center	Chart review	Academic, single-center	November 2022–March 2024	Overall population: 9.5 Patients switching to less-frequent dosing: 6.4	86	ORR, DOR, PFS, time to response

Abbreviations: AE, adverse event; CRS, cytokine release syndrome; DOR, duration of response; EMR, electronic medical record; ICANS, immune effector cell-associated neurotoxicity syndrome; IMF, International Myeloma Foundation; IVIG, intravenous immunoglobulin; LOS, length of stay; mFU, median follow-up; MM, multiple myeloma; NR, not recorded; PFS, progression-free survival; ORR, overall response rate; OS, overall survival; TCZ, tocilizumab; TTNT, time to next treatment; US, United States; USMIRC, US Myeloma Innovations Research Collaborative.

**Table 2:** Summary of patient characteristics in studies with ≥50 patients and ≥3 months median follow-up.

Study ID	Overall/Subgroup Details	Sample Size	Median Age (Range), Years	Female, n (%)	Race, n (%)			High - Risk Cytogenetics, n (%)	Median Prior Lines of Therapy	Extramedullary Disease, n (%)
					White	Black	Asian			
Banerjee 2023b [25,26]	Overall	247	69 (41–89)	111 (44.9)	138 (75.8) ^a^	23 (12.6) ^a^	21 (11.5) ^a^	--	--	14 (5.7)
Dima 2023 [12,27,28,29,30]	Overall	106	66.5 (35–87)	55 (54) ^b^	72 (68)	28 (26)	2 (2)	56 (59)	6	45 (42)
Firestone 2023 [17,31,32,33]	Overall	52	70 (30–80)	--	--	--	--	17 (33)	7	18 (35)
Mohan 2024 [19,34]	Overall	110	68 (37–89)	54 (49)	67 (61)	32 (29)	2 (1.8)	59 ^c^ (62)	6	48 (44)
Pianko 2024 [35]	Overall	419	65 (58–73) ^d^	183 (43.7)	185 (63.4) ^e^	91 (31.2) ^e^	16 (5.4) ^e^	--	5	--
Riedhammer 2024 [20,36]	Overall	123	67 (35–87)	53 ^f^ (43.1)	--	--	--	39 (36.8) ^g^	6	43 (36.1)
Tan-Asoori 2023 [37,38,39]	Overall	204	66 (33–91)	91 (45)	143 (70)	15 (7)	19 (9)	90 (44)	6	38 (19)
Tan 2024 [40,41]	Overall	86	71 (64–78) ^d^	44 (51)	65 (76)	14 (16)	--	56 (71) ^h^	6	30 (38) ^h^

Notes: Double dashes (“—”) indicate data not reported. ^a^ Evaluated population = 186; ^b^ calculated from the reported proportion of males; ^c^ n = 95 evaluable; ^d^ IQR; ^e^ evaluable population, n = 292; ^f^ n hand calculated; ^g^ evaluable population, n = 106; ^h^ evaluable population, n = 79. Abbreviations: EMD, extramedullary disease; IQR, interquartile range.

**Table 3:** Summary of MajesTEC-1 ineligibility in studies with ≥50 patients and ≥3 months median follow-up.

Study ID	Overall/Subgroup Details	Sample Size	MajesTEC-1 Ineligible Patients, n (%)	Prior BCMA Therapy, n (%)	ECOG PS ≥2, n (%)	Cytopenia, n (%)				Renal Impairment/Failure, n (%)	CrCl <30 mL/min or 40 mL/min, n (%)
						Overall	Anemia	Neutropenia	Thrombocytopenia		
Banerjee 2023b [25,26]	Overall	247	--	48 (19.4)	--	--	126 (51.0)	55 (22.3)	--	100 (40.5)	--
Dima 2023 [12,27,28,29,30]	Overall	106	88 (83)	56 (53)	35 (33)	33 (31)	27 (25)	2 (2)	21 (20)	14 (13)	14 (13)
Firestone 2023 [17,31,32,33]	Overall	52	--	27 (52)	--	--	--	--	--	--	--
Mohan 2024 [19,34]	Overall	110	--	38 (35)	--	--	--	--	--	--	--
Pianko 2024 [35]	Overall	419	--	102 (24.3)	--	--	164 (39.1)	50 (11.9)	--	206 (49.2)	--
Riedhammer 2024 [20,36]	Overall	123	48 ^a^ (39)	45 (37.4)	--	--	--	--	--	--	
Tan-Asoori 2023 [37,38,39]	Overall	204	122 (70) ^b^	91 (45.0)	--	--	--	--	--	--	25 (12)
Tan 2024 [40,41]	Overall	86	--	32 (37)	5 (10) ^c^	--	--	--	--	--	9 (10)

Notes: Double dashes (“—”) indicate data not reported. ^a^ n hand calculated; ^b^ based on the evaluated population (n = 175); ^c^ based on the evaluable population, n = 50. Abbreviations: BCMA, B-cell maturation antigen; CrCl, creatinine clearance; ECOG, Eastern Cooperative Oncology Group performance status; EMD, extramedullary disease.

**Table 4:** Summary of key effectiveness outcomes in studies with ≥50 patients and ≥3 months median follow-up.

Study ID	Overall/Subgroup Details	Timepoint/mFU	Response Evaluable Population Size	Overall Response Rate, n (%)			Survival Evaluable Population Size	PFS		OS	
				PR or Better	VGPR or Better	CR or Better		Median, Months (95% CI)	6-Month Rate, % (95% CI)	Median, Months (95% CI)	6-Month Rate, % (95% CI)
Dima 2023 [12,27,28,29,30]	Overall	Median of 3.8 months	104	70 ^a^ (66)	49 (46) ^b^	31 (29) ^b^	106	5.4 (3.4, NR)	--	NR	70 (61, 80)
	>70 years old	Median of 3.8 months ^c^	34	24 (71)	--	10 (30) ^d^	33	5.4 (2.8, NR)	--	--	--
Firestone 2023 [17,31,32,33]	Overall	Median of 3.1 months	47	30 (64)	18 ^a^ (38)	--	52	NR	--	--	--
	Prior anti-BCMA exposure	Median of 3.1 months ^c^	26	13 (50.0)	--	--	27	3.4	--	--	--
Mohan 2024 [19,34]	Overall	Median of 3.5 months	98	61 (62)	50 ^a^ (51)	20 ^a^ (20)	110	NR	52 (42, 64)	NR	80 (72, 89)
Riedhammer 2024 [20,36]	Overall	Median of 5.5 months	123	73 ^a^ (59.3)	59 ^a^ (48.0) ^b^	27 ^a^ (22.0) ^b,e^	123	8.7	--	NR	--
Tan-Asoori 2023 [37,38,39]	Overall	Median of 5 months	180	115 ^a^ (64)	90 ^a^ (50) ^b^	34 ^a^ (19) ^b^	204	13	57.7	15	76.2
Tan 2024 [40,41]	Overall	Median of 9.5 months	77	47 (61)	33 (43)	--	86	--	52.4 (42.4, 64.7)	--	--
	Prior BCMA-directed therapy	Median of 6.4 months	32	14 (43)	--	--	32	--	90.0 (79.1, 100.0)	--	--

Notes: Double dashes (“—”) indicate data not reported. ^a^ n hand calculated; ^b^ calculated from stratified best response; ^c^ median follow-up assumed to apply to subgroup data, as both share the same data cut-off; ^d^ % hand calculated; ^e^ reported as near complete or complete response. Abbreviations: BCMA, B-cell maturation antigen; CI, confidence interval; CR, complete response; mFU, median follow-up; NR, not reached; PR, partial response; VGPR, very good partial response.

**Table 5:** Summary of key safety outcomes in studies with ≥50 patients and ≥3 months median follow-up.

Study ID	Overall/Subgroup Details	Sample Size	Timepoint/mFU	CRS, n (%)				ICANS, n (%)				Infections, n (%)	
				Any Grade	Grade 1	Grade 2	Grade 3+	Any Grade	Grade 1	Grade 2	Grade 3+	Any Grade	Grade 3+
Chart review—mixed inpatients and outpatient, or not reported													
Mohan 2024 [19,34]	Overall	110	Median of 3.5 months	62 ^a^ (56)	57 ^a^ (51.8) ^b,c^		5 (4.5) ^b^	12 (11)	--	--	5 (4.5)	44 (40)	29 (26)
Riedhammer 2024 [20,36]	Overall	123	Median of 5.5 months	72 (58.5)	--	--	2 (1.6)	--	--	--	--	67 (54.5)	33 (26.8)
Tan-Asoori 2023 [37,38,39]	Overall	204	Median of 5 months	110 (53.9) ^a,b^	84 (41.2) ^a,b^	25 (12.3) ^a,b^	1 (0.5) ^a,b^	--	--	--	--	115 (60.0)	--
Chart Review—inpatient monitoring													
Dima 2023 [12,27,28,29,30]	Overall	106	Median of 3.8 months	68 (64)	57 (54)	10 (9)	1 (1)	15 (14)	5 (5)	7 (6)	3 (3)	33 (31)	--
	>70 years old	33	July 2023 cut-off	22 (67)	--	--	1 (3)	7 (21)	--	--	0 (0)	11 (33)	--
Firestone 2023 [17,31,32,33]	Overall	52	Median of 3.1 months	27 (52)	--	--	--	--	--	--	--	--	--
Tan-Asoori 2023 [37,38,39]	Inpatients	160	Median of 5 months	94 (59)	72 (45)	22 (14)	0 (0)	--	--	--	--	--	--
Chart review—outpatient monitoring													
Tan-Asoori 2023 [37,38,39]	Outpatients	44	Median of 5 months	16 (36)	12 (27)	3 (7)	1 (2)	--	--	--	--	--	--
Secondary databases													
Banerjee 2023b [25,26]	Overall	76	Median of 5.1 months	14 (18.4)	8 (10.5)	3 (3.9)	1 (1.3)	3 (3.9)	2 (2.6)	1 (1.3)	0 (0)	--	--

Notes: Double dashes (“—”) indicate data not reported. Results are based on the most recent timepoint in each study. ^a^ n hand calculated; ^b^ % hand calculated; ^c^ reported as grade 1 and 2. Abbreviations: CRS, cytokine release syndrome; ICANS, immune effector cell-associated neurotoxicity syndrome; mFU, median follow-up.

## Data Availability

All data generated or analyzed during this study are included in this published article and its Supplementary Information files.

## Supplementary Materials

The following supporting information can be downloaded at: https://www.mdpi.com/article/10.3390/cancers17071235/s1, Document S1: SLR Protocol; Document S2: PRISMA 2020 checklist. References [2,3,4,5,6,13,14,15,54,55,56] are cited in the supplementary materials.

## Appendix A. PICOS Criteria

**Table A1:** Eligibility criteria for the systematic literature review.

Criteria	Inclusion	Exclusion
Population	Adult (≥18 years) patients with multiple myeloma	Patients aged < 18 years
Interventions	Teclistamab	Interventions not listed
Comparators	No restrictions	--
Outcomes	No restrictions	--
Study design	Real-world observational studies (prospective, retrospective)	Clinical trials Case reports or case series Pooled analyses of trials Systematic literature reviews ^a^ Non-systematic/narrative reviews
Publication type	Full-text publications Conference abstracts/posters ^b^	Letters to editors Editorials Commentary Expert opinion Guidelines
Language	Only studies published in English	Studies published in a language other than English (even if the abstract is in English)
Time	Published between 1 January 2023 and 30 June 2024	Published before 2023

Notes: Double dashes (“—”) indicate data not reported. ^a^ Systematic reviews were excluded, but the bibliography of relevant systematic reviews was reviewed to capture relevant citations; ^b^ conference abstract/poster citations captured through a search of Embase were screened, and conferences of interest were also searched separately in the Northern Light database or published proceedings from target conferences.

## Appendix B. Literature Search Strategies

**Table A2:** Search strategy for Embase.

Database: Embase 1974 to 22 May 2024 Search Executed on 23 May 2024			
#	Criteria	Search Terms	Results
1	Population terms	exp multiple myeloma/	102,883
2		exp plasmacytoma/	14,274
3		exp Paraproteinemias/	183,815
4		(myeloma or (multiple adj2 myeloma$) or plasmacytom$ or plasmocytom$ or mgus or (monoclonal adj2 gammopath$)).mp.	149,247
5		or/1–4	221,071
6	Intervention terms	exp teclistamab/	406
7		(teclistamab or Tecvayli or JNJ-64007957 or teclistamab-cqyv).mp.	424
8		or/6–7	424
9	SIGN filters for observational studies	Clinical study/	166,793
10		Case control study/	217,895
11		Family study/	25,824
12		Longitudinal study/	213,746
13		Retrospective study/	1,624,202
14		Prospective study/	919,580
15		Randomized controlled trials/	274,328
16		14 not 15	908,367
17		Cohort analysis/	1,167,616
18		(Cohort adj (study or studies)).mp.	517,217
19		(Case control adj (study or studies)).tw.	176,157
20		(follow up adj (study or studies)).tw.	76,223
21		(observational adj (study or studies)).tw.	276,600
22		(epidemiologic$ adj (study or studies)).tw.	125,861
23		(cross sectional adj (study or studies)).tw.	373,627
24		(retrospective adj7 (study or studies or design or analysis or analyses or cohort or data or review)).ti,ab.	1,254,000
25		or/9–13,16–24	4,466,453
26	Combined criteria	5 and 8 and 25	96
27	Language filter	limit 26 to english language	96
28	Date filter	limit 27 to yr=“2023-Current”	72

Note: An Embase search was performed again at the end of June 2024 and no additional, relevant publications were identified.

**Table A3:** Search strategy for MEDLINE.

Database: Ovid MEDLINE(R) ALL <1946 to 22 May 2024> Search Executed on 23 May 2024			
#	Criteria	Search Terms	Results
1	Population terms	exp multiple myeloma/	49,135
2		exp plasmacytoma/	8932
3		exp Paraproteinemias/	64,055
4		(myeloma or (multiple adj2 myeloma$) or plasmacytom$ or plasmocytom$ or mgus or (monoclonal adj2 gammopath$)).mp.	84,636
5		or/1–4	96,987
6	Intervention terms	(teclistamab or Tecvayli or JNJ-64007957 or teclistamab-cqyv).mp.	89
7	SIGN filters for observational studies	Epidemiologic studies/	9543
8		exp case control studies/	1,506,567
9		exp cohort studies/	2,607,741
10		Case control.tw.	162,188
11		(cohort adj (study or studies)).tw.	351,806
12		Cohort analy$.tw.	13,035
13		(Follow up adj (study or studies)).tw.	58,204
14		(observational adj (study or studies)).tw.	178,369
15		Longitudinal.tw.	345,820
16		Retrospective.tw.	812,750
17		Cross sectional.tw.	562,700
18		Cross-sectional studies/	502,656
19		or/7–18	4,003,471
20	Combined criteria	5 and 6 and 19	11
21	Language filter	limit 20 to english language	11
22	Date filter	limit 21 to yr=“2023-Current”	11

**Table A4:** Search strategy for Northern Light Life Sciences Conference Abstracts.

Database: Northern Light Life Sciences Conference Abstracts 2010–2024 Week 20 Search Executed on 23 May 2024			
#	Criteria	Search Terms	Results
1	Population terms	exp multiple myeloma/	27,388
2		exp plasmacytoma/	1694
3		exp Paraproteinemias/	30,424
4		(myeloma or (multiple adj2 myeloma$) or plasmacytom$ or plasmocytom$ or mgus or (monoclonal adj2 gammopath$)).mp.	30,055
5		or/1–4	32,556
6	Intervention terms	(teclistamab or Tecvayli or JNJ-64007957 or teclistamab-cqyv).mp.	102
7	Conference filter	Academy of Managed Care Pharmacy.cf.	2749
8		American Society of Clinical Oncology.cf.	79,140
9		American Society of Hematology.cf.	66,037
10		European Hematology Association.cf.	31,727
11		European Society for Medical Oncology.cf.	22,763
12		International Myeloma.cf.	2497
13		Oncology Nursing Society.cf.	1267
14		Society of Hematologic Oncology.cf.	2052
15		or/7–14	208,232
16	Combined criteria	5 and 6 and 15	82
17	Date filter	limit 16 to yr=“2023-Current”	47

## Appendix C. List of Conferences Searched

**Table A5:** Conferences searched in the systematic literature review.

Conference	Dates Held	Search Method
Academy of Managed Care Pharmacy (AMCP) Meeting	21–24 March 2023 15–18 April 2024	Northern Light database via Ovid Hand search of website
AMCP Nexus	16–19 October 2023	Hand search of website
American Society for Transplantation and Cellular Therapy (ASTCT) and Center for International Blood and Marrow Transplant Research (CIBMTR) Tandem Meeting	15–19 February 2023 21–24 February 2024	Hand search of website
American Society of Clinical Oncology (ASCO) Annual Meeting	2–6 June 2023 31 May–4 June 2024	Northern Light database via Ovid Hand search of website
American Society of Hematology (ASH) Annual Meeting	9–12 December 2023	Hand search of website
European Hematology Association (EHA) Annual Congress	8–11 June 2023 13–16 June 2024	Northern Light database via Ovid Hand search of website
European Society for Medical Oncology (ESMO)	20–24 October 2023	Northern Light database via Ovid
European Myeloma Network (EMN) Meeting	20–22 April 2023 18–20 April 2024	Hand search of website
Hematology/Oncology Pharmacy Association (HOPA) Annual Conference	29 March–1 April 2023 3–6 April 2024	Hand search of website
International Conference on Oncology and Research Treatment (ORT)	30 November–1 December 2023	Hand search of website
International Myeloma Society (IMS) Annual Meeting	27–30 September 2023	Northern Light database via Ovid
Journal of the Advanced Practitioner in Oncology (JADPRO) Live	9–12 November 2023	Hand search of website
Lymphoma, Leukemia & Myeloma (LL&M) Congress	18–21 October 2023	Hand search of website
Oncology Nursing Society (ONS) Congress	26–30 April 2023 24–28 April 2024	Northern Light database via Ovid Hand search of website
Society of Hematologic Oncology (SOHO) Annual Meeting	6–9 September 2023	Northern Light database via Ovid

## Appendix D. Risk of Bias Assessment

**Table A6:** Newcastle–Ottawa quality assessment scale—cohort studies.

Domain	Response
Selection	
1. Representativeness of the exposed cohort	Truly representative of the average _______________ (describe) in the community * Somewhat representative of the average ______________ in the community * Selected group of users (e.g., nurses, volunteers) No description of the derivation of the cohort
2. Selection of the non-exposed cohort	Drawn from the same community as the exposed cohort * Drawn from a different source No description of the derivation of the non-exposed cohort
3. Ascertainment of exposure	Secure record (e.g., surgical records) * Structured interview * Written self-report No description
4. Demonstration that the outcome of interest was not present at the start of the study	Yes * No
Comparability	
1. Comparability of cohorts on the basis of the design or analysis	Study controls for _____________ (select the most important factor) * Study controls for any additional factor (this criterion could be modified to indicate specific control for a second important factor) *
Outcomes	
1. Assessment of outcome	Independent blind assessment * Record linkage * Self-report No description
2. Was the follow-up long enough for the outcomes to occur?	Yes (select an adequate follow-up period for the outcome of interest) * No
3. Adequacy of the follow-up of cohorts	Complete follow up—all subjects accounted for * Subjects lost to follow up unlikely to introduce bias—small number lost—>____% (select an adequate %) follow up, or description provided of those lost) * Follow up rate <____% (select an adequate %) and no description of those lost No statement

Note: A study can be awarded a maximum of one star (*) for each numbered item within the selection and exposure categories. A maximum of two stars can be given for comparability.

## Appendix E. Summary Tables of All the Included Studies

**Table A7:** Summary of study characteristics and outcomes evaluated.

Study ID	Study Design	Data Source	Chart Review vs. Secondary Database	Setting	Study Timeframe	mFU (Months)	Sample Size	Relevant Outcomes Evaluated
Asoori 2023 [57]	Retrospective	Patients treated at UCSF	Chart review	Academic, single-center	NR—July 2023	3	46	ORR, OS, PFS, infection AEs, IVIG use in AEs
Banerjee 2023a [44]	Retrospective	All-payer claims database (ARMMRD)	Secondary database	Multi-center	October 2022–July 2023	--	182	LOS, CRS
Banerjee 2023b [25,26]	Retrospective	Acentrus MM electronic medical records (EMRs)	Secondary database	Academic centers/community-based hospitals, multi-center	October 2022–November 2023	5.1	247	LOS, CRS, ICANS, step-up dosing schedule, TTNT, or death
Bansal 2023 [45,58]	Retrospective	Patients treated at the Mayo Clinic, Rochester, MN	Chart review	Academic, single-center	October 2020–July 2023	--	24	LOS, CRS, remote monitoring, hospitalizations
Bansal 2024 [59]	Retrospective	Patients treated at the Mayo Clinic Rochester, MN	Chart review	Academic, single-center	December 2022–January 2024	--	48	ORR
Bolton 2024 [60]	Prospective	Patients treated at three Kaiser Permanente institutions	Chart review	Academic, multi-center	NR	--	9	CRS, ICANS, discontinuations due to any cause
Catamero 2023 [61]	Retrospective	Patients treated at the Mount Sinai Hospital	Chart review	Academic, single-center	December 2022–May 2023	--	26	CRS, TCZ use in AE management
Charkviani 2024 [62]	Retrospective	Patients treated at the Mayo Clinic Healthcare System	Chart review	Academic, multi-center	December 2022–May 2023	--	34	Acute kidney injury (AKI) incidence and treatment
Dima 2023 [12,27,28,29,30]	Retrospective	Patients treated at USMIRC centers	Chart review	Academic, multi-center	August 2022–August 2023	3.8	106	CRS, ICANS, infections, ORR, DOR, OS, PFS, TCZ use in AE management, hospitalizations
Faiman 2023 [63]	Retrospective	Patients treated at Cleveland Clinic	Chart review	Academic, single-center	December 2022–May 2023	2.5	26	CRS, TCZ use in AE management, ICANS, infections, best response
Firestone 2023 [17,31,32,33]	Retrospective	Patients treated at the Memorial Sloan Kettering Cancer Center	Chart review	Academic, single-center	November 2022–July 2023	3.1	52	Survival, PFS, ORR, safety
Ghamsari 2024 [64]	Retrospective	Patients treated at the University of California San Diego	Chart review	Academic, single-center	January 2023–June 2023	--	18	CRS, ICANS, infections, ORR, DOR, OS, PFS, TCZ use in AE management
Glenn 2024 [65]	Prospective	Patients treated at the Smilow Cancer Hospital at Yale New Haven Hospital	Chart review	Academic, single-center	NR	--	18	CRS
Gong 2023 [21]	Retrospective	FDA Adverse Event Reporting System database	Secondary database	Multi-center	2019–2023	--	719 ^a^	Reporting OR, CRS, ICANS, infection, non-ICANS neurotoxicity, mortality, hospitalizations
Gordon 2023 [66]	Retrospective	Patients treated at four New York City metro area centers	Chart review	Academic, multi-center	NR	--	58	ORR, CRS, ICANS, tocilizumab use in AE management, discontinuations
Graf 2024 [67]	Retrospective	Patients treated at the Medical University of South Carolina	Chart review	Academic, single-center	November 2022–August 2023	--	25	Hospitalization, LOS, proportion of fever at each dose of teclistamab, incidence, severity, and onset of CRS
Grajales-Cruz 2023 [68,69]	Retrospective	Patients treated at the Lee Moffitt Cancer Center; chart review	Chart review	Academic, single-center	December 2022–October 2023	4.2	36	ORR, PFS, OS, CRS, TCZ use in AE management, infection, LOS, ICU admission
Hamadeh 2024 [18,70,71]	Retrospective	Memorial Sloan Kettering Cancer Center’s institutional plasma cell disorders database	Chart review	Academic, single-center	November 2022–July 2023	--	69	CRS
Hebraud 2023 [51]	Retrospective	Patients treated at Institut Universitaire du Cancer de Toulouse	Chart review	Academic, single-center	January 2021–July 2023	--	8	CRS, ORR, TCZ, and IVIG use in AE management
Howard 2023 [47]	Retrospective	Patients treated at the Memorial Sloan Kettering Cancer Center	Chart review	Academic, single-center	NR	--	23	LOS, unscheduled physician communications, hospitalizations
Kawasaki 2024 [72]	Retrospective	Patients treated at the University of California Davis (UCD) medical center	Chart review	Academic, single-center	December 2022–December 2023	--	27	CRS, ICANS, LOS, hematological toxicities, infections, hepatotoxicity, diarrhea, and IVIG in AE management
Kowalski 2023 [42]	Prospective	Patients treated at the University of Miami Hospital & Clinics, Sylvester Comprehensive Cancer Center	Chart review	Academic, single-center	October 2022–July 2023	3.4 ^b^	31	Best response, ORR, CRS, ICANS, PFS
Kumar 2023 [73]	Prospective	Patients treated at the University of Connecticut Health Center	Chart review	Academic, single-center	November 2022–February 2023	--	9	Best response, ORR, CRS, ICANS
Lachenal 2023 [74]	Retrospective	Patients treated in the French AP program	Secondary database ^c^	Multi-center	November 2022–October 2023	5.2	15	CRS, TCZ use in AE management, ICANS, ORR, IVIG in AE management
Marin 2023 [43]	Prospective	Patients treated at the Emory University hospital	Chart review	Academic, single-center	December 2022–August 2023	--	53	CRS, TCZ use in AE management, TCZ dosing, ICANS, readmissions,
Midha 2023 [22]	Retrospective	Patients treated at the Dana–Farber Cancer Institute/Brigham and Women’s Hospital	Chart review	Academic, single-center	NR—August 2023	2.3 ^b^	56	CRS, TCZ use in AE management, infections, ORR, PFS
Mohan 2024 [19,34]	Retrospective	Patients treated at five US academic centers	Chart review	Academic, multi-center	NR	3.5	110	CRS, ICANS, infections, ORR, best response, LOS, IVIG use in AE management, TCZ use in AE management
Mooney 2024 [46]	Retrospective	Patients treated at the Sidney Kimmel Comprehensive Cancer Center at Johns Hopkins Hospital	Chart review	Academic, single-center	January 2023—NR	--	19	Hospital admission, LOS, CRS, and ICANS
Nader 2023 [23]	Retrospective	Patients treated at the Indiana University School of Medicine ^d^	Chart review	Academic, single-center ^d^	2000–2021 ^e^	33.6 ^f^	49	PFS, OS, treatment response
Perrot 2023 [75]	Retrospective	Patients treated in the French AP program	Secondary database ^c^	Multi-center	October 2022–April 2023	2.9	572	Discontinuations, AE-related mortality
Pianko 2024 [35]	Retrospective	Komodo Healthcare Map^TM^	Secondary database	Multi-center	October 2022–December 2023	4.2	419	Dosing schedule of teclistamab, time to less frequent dosing, TTNT
Rees 2024 [76]	Retrospective	Patients treated at the Mayo Clinic centers	Chart review	Academic, multi-center	April 2018–June 2023	9	41	PFS, DOR, OS
Riedhammer 2024 [20,36]	Retrospective	Patients treated at 18 German centers	Chart review	Multi-center	July 2022–October 2023	5.5	123	Time to response, best response, ORR, PFS, infections
Sandahl 2023 [7,77]	Retrospective	Patients treated at three Mayo Clinic centers	Chart review	Academic, multi-center	October 2022–September 2023	--	49	CRS, ICANS, TCZ use in AE management, admissions, LOS, time between TEC administration and check-out
Schaefers 2023 [78]	Prospective	Patients treated at the University Medical Center Hamburg-Eppendorf tertiary center	Chart review	Academic, single-center	July 2022–May 2023	3.4 ^b^	16	CRS, ICANS, infections, neutropenia, ORR, OS, PFS, DOR
Tabbara 2024 [79]	Retrospective	Patients treated at the Johns Hopkins Sidney Kimmel Comprehensive Cancer Center; Inpatient/Outpatient unit	Chart review	Academic, single-center	January 2023–December 2023	--	25	Neutropenia, infection, CRS, ICANS, patients receiving dexamethasone, hospital admission, death
Tan-Asoori 2023 [37,38,39]	Retrospective	Patients treated at IMF-associated centers	Chart review	Academic, multi-center	NR—October 2023	5	204	ORR, OS, PFS, CRS, ICANS, TCZ use in AE management, IVIG use in AE management, LOS
Tan 2023 [53,80]	Retrospective	Premier healthcare database	Secondary database	Multi-center	November 2022–March 2023	--	113	LOS, step-up dosing schedule, hospitalizations, CRS, TCZ use in AEs
Tan 2024 [40,41]	Retrospective	Patients treated at the Memorial Sloan Kettering Cancer Center	Chart review	Academic, single-center	November 2022–March 2024	Overall population: 9.5 Patients switching to less-frequent dosing: 6.4	86	ORR, DOR, PFS, time to response
Varshavsky-Yanovsky 2023 [9,81]	Retrospective	Patients treated at Fox Chase Cancer Center	Chart review	Academic, single-center	December 2022–May 2023	--	18	CRS, ICANS
Venkatesh 2023 [82]	Retrospective	Patients treated at the University of Kansas	Chart review	Academic, single-center	NR—February 2023	3.1	22	CRS, ICANS, ORR, mortality, best response

Notes: ^a^ Number of adverse event cases; note that this differs from other studies which report the number of individual patients and which, therefore, were not included in the result summary; ^b^ converted from days to months; ^c^ the French AP program follows hospitals that request reimbursement for innovative medicines prior to and after market authorization, with data coming from these reimbursement claims; ^d^ not reported and assumed based on all but one authors’ affiliation being the Indiana University School of Medicine; ^e^ given the FDA approval of TEC in 2022, the year 2000 as the start date is assumed to be an error; ^f^ converted from years to months. Abbreviations: AE, adverse event; AKI, acute kidney injury; AP, Accès Précoce; ARMMRD, all-payer real-world multiple myeloma research-ready data; CRS, cytokine release syndrome; DOR, duration of response; EMR, electronic medical record; FDA, Food and Drug Administration; ICANS, immune effector cell-associated neurotoxicity syndrome; ICU, intense care unit; IMF, International Myeloma Foundation; IVIG, intravenous immunoglobulin; LOS, length of stay; mFU, median follow-up; MM, multiple myeloma; MN, Minnesota; NR, not recorded; PFS, progression-free survival; ORR, overall response rate; OS, overall survival; TCZ, tocilizumab; TEC, teclistamab; TTNT, time to next treatment; UCD, University of California Davis; UCSF, University of California San Francisco; US, United States; USMIRC, US Myeloma Innovations Research Collaborative.

**Table A8:** Summary of patient characteristics.

Study ID	Overall/Subgroup Details	Sample Size	Median Age (Range), Years	Female, n (%)	Race, n (%)			High Risk Cytogenetics, n (%)	Median Prior Lines of Therapy	Extramedullary Disease, n (%)
					White	Black	Asian			
Asoori 2023 [57]	Overall	46	71 (50–91)	23 (50.0) ^a^	23 (50.0)	2 (4.3)	14 (30.4)	13 (38.2)	7	--
Banerjee 2023a [44]	Overall	182	73 (39–85)	91 (50.0)	123 (67.6)	20 (11.0)	5 (2.7)	--	--	--
Banerjee 2023b [25,26]	Overall	247	69 (41–89)	111 (44.9)	138 (75.8) ^b^	23 (12.6) ^b^	21 (11.5) ^b^	--	--	14 (5.7)
Bansal 2023 [45,58]	Overall	24	66	9 (39) ^a^	--	--	--	9 (38)	5	--
Bansal 2024 [59]	TEC treatment group ^c^	48	--	--	--	--	--	--	--	--
Bolton 2024 [60]	Overall	9	--	--	--	--	--	--	--	--
Catamero 2023 [61]	Overall	26	--	--	--	--	--	--	--	--
Charkviani 2024 [62]	TEC treatment group	34	66.4 (55.4–70.8) ^d^	11 (32.4)	31 (91.2)	1 (2.9)	1 (2.9)	--	--	--
Dima 2023 [12,27,28,29,30]	Overall	106	66.5 (35–87)	55 (54) ^a^	72 (68)	28 (26)	2 (2)	56 (59)	6	45 (42)
	>70 years old	33	75 (71–87)	--	--	--	--	19 (58)	6	13 (39)
	Patients with EMD	45	68 (43–83)	10 (22.0) ^a^	35 (78.0)	--	--	25 (55.5)	6	45 (100.0)
Faiman 2023 [63]	Overall	26	64 (45–75)	14 ^e^ (54)	19 ^e^ (73)	--	--	--	7	--
Firestone 2023 [17,31,32,33]	Overall	52	70 (30–80)	--	--	--	--	17 (33)	7	18 (35)
Ghamsari 2024 [64]	Overall	18	67 (50–83)	--	--	--	--	11 (84.6) ^f,g^	6.5	8 (44)
Glenn 2024 [65]	Overall	18	--	--	--	--	--	--	--	--
Gong 2023 [21]	Overall	719 cases ^h^	65 (57–72) ^e^	229 (31.8)	--	--	--	--	--	--
Gordon 2023 [66]	Overall	58	67 (45–88)	31 (53.4)	26 (45.6)	15 (26.3)	3 (5.2)	21 (36.2)	5	28 (48.3)
Graf 2024 [67]	Overall	25	66 (37–78)	12 (48.0)	14 (56.0)	--	--	9 (36.0)	5	13 (52.0)
Grajales-Cruz 2023 [68,69]	Overall	36	67 (48–88)	16 (44.4) ^a^	--	--	--	15 (44.1)	7	16 (57.0)
Hamadeh 2024 [18,70,71]	Overall	72	(39–88)	30 (41.7) ^i^	56 (77.8) ^i^	12 (16.7) ^i^	--	36 (50.0) ^i^	--	21 (29.2) ^i^
	Prior T-cell redirection therapies (TCRT)	27	70 (51–88)	10 (37)	23 (85)	2 (7)	--	19 (70)	8	9 (33)
	No prior TCRT	45	69 (39–88)	20 (44)	33 (73)	10 (22)	--	17 (38)	5	12 (27)
Hebraud 2023 [51]	Overall	8	69 (43–81)	--	--	--	--	--	4	--
Howard 2023 [47]	Overall	23	67 (51–88)	--	--	--	--	5 (22)	6	6 (26)
Kawasaki 2024 [72]	Overall	27	--	12 (44.4) ^i^	18 (66.7) ^i^	--	--	--	--	8 (29.6) ^i^
	Dosing schedule 1, 3, 5 (days)	23	69	9 (39) ^f^	16 (69)	--	--	--	--	6 (26)
	Dosing schedule 1, 4, 7 (days)	4	64	3 (75) ^f^	2 (50)	--	--	--	--	2 (50)
Kowalski 2023 [42]	Overall ^j^	31	71 (50–84)	21 (68)	22 (71)	--	--	9 (29)	5	24 (77)
Kumar 2023 [73]	Overall	9	75 (41–81)	--	--	--	--	--	6	--
Lachenal 2023 [74]	Overall	15	68 (58–83)	--	--	--	--	7 (70.0) ^k^	4	--
Marin 2023 [43]	Overall	53	69 (43–83)	15 (28.3) ^a,i^	25 (47.2) ^i^	24 (45.3) ^i^	1 (1.9) ^i^	21 (39.6) ^i^	--	--
	No prophylactic tocilizumab (TCZ)	15	58 (47–73)	2 (13.3) ^a^	7 (46.7)	8 (53.3)	--	4 (26.7)	6	--
	Prophylactic TCZ	38	69 (43–83)	13 (34.2) ^a^	18 (47.4)	16 (42.1)	1 (2.6)	17 (44.7)	5	--
Midha 2023 [22]	Overall	56	69 (45–83)	21 (37.5) ^a^	--	--	--	--	6	24 (42.9)
Mohan 2024 [19,34]	Overall	110	68 (37–89)	54 (49)	67 (61)	32 (29)	2 (1.8)	59 ^l^ (62)	6	48 (44)
Mooney 2024 [46]	Overall	19	--	--	--	--	--	--	--	--
Nader 2023 [23]	Overall	49	70 (59–75) ^d^	27 (55.0)	41 (84.0)	5 (10.0)	--	--	>4	--
Perrot 2023 [75]	Overall	572	71 (64–76) ^d^	241 (42.1)	--	--	--	124 (21.7)	4	121 (21.2)
Pianko 2024 [35]	Overall	419	65 (58–73) ^d^	183 (43.7)	185 (63.4) ^m^	91 (31.2) ^m^	16 (5.4) ^m^	--	5	--
Rees 2024 [76]	TEC treatment group ^c^	41	--	--	--	--	--	--	--	--
Riedhammer 2024 [20,36]	Overall	123	67 (35–87)	53 ^e^ (43.1)	--	--	--	39 (36.8) ^n^	6	43 (36.1)
Sandahl 2023 [7,77]	Overall	49	67.2 (38.7–84.2)	18 (36.7)	43 (87.8)	3 (6.1)	1 (2.0)	31 (63.3)	--	3 (6.1)
Schaefers 2023 [78]	Overall	16	65.5 (51–86)	5 (31)	--	--	--	8 (80)	6	--
Tabbara 2024 [79]	Overall	25	70 (59–89)	14 (56.0)	19 (76.0)	5 (20.0)	0 (0.0)	--	6	--
Tan-Asoori 2023 [37,38,39]	Overall	204	66 (33–91)	91 (45)	143 (70)	15 (7)	19 (9)	90 (44)	6	38 (19)
Tan 2023 [53,80]	Overall	113	65 (58–74) ^d^	44 (38.9)	74 (65.5)	24 (21.2)	3 (2.7)	--	--	--
Tan 2024 [40,41]	Overall	86	71 (64–78) ^d^	44 (51)	65 (76)	14 (16)	--	56 (71) ^o^	6	30 (38) ^o^
	Patients switched to a less frequent dosing schedule	32	70 (65–78) ^d^	20 (62) ^a^	--	3 ^e^ (9)	--	17 (59)	6	10 (34) ^p^
Varshavsky-Yanovsky 2023 [9,81]	Overall	18	66 (46–81)	--	--	--	--	--	--	--
Venkatesh 2023 [82]	Overall	22	70 (43–85)	9 (41) ^g^	--	--	--	9 (41)	6	14 (64)

Notes: Double dashes (“—”) indicate data not reported. ^a^ Calculated from the reported proportion of males; ^b^ evaluated population = 186; ^c^ overall population of combined multiple treatments, with TEC-only data limited; ^d^ IQR; ^e^ n hand calculated; ^f^ evaluable population n = 13; ^g^ % hand calculated; ^h^ number of individual cases; ^i^ calculated from stratified cohorts; ^j^ the overall population was treated with prophylactic tocilizumab; ^k^ n = 10 evaluable; ^l^ n = 95 evaluable; ^m^ evaluable population n = 292; ^n^ evaluable population n = 106; ^o^ evaluable population n = 79; ^p^ evaluable population n = 29. Abbreviations: EMD, extramedullary disease; IQR, interquartile range; TCRT, T-cell redirection therapies; TCZ, tocilizumab; TEC, teclistamab.

**Table A9:** Summary of MajesTEC-1 ineligibility.

Study ID	Overall/Subgroup Details	Sample Size	MajesTEC-1 Ineligibility, n (%)	Prior BCMA Therapy, n (%)	ECOG PS ≥2, n (%)	Cytopenia, n (%)				Renal Impairment/Failure, n (%)	CrCl < 30 mL/min or 40 mL/min, n (%)
						Overall	Anemia	Neutropenia	Thrombocytopenia		
Asoori 2023 [57]	Overall	46	31 (67.3)	16 (34.8)	--	--	--	--	--	--	15 (32.6)
Banerjee 2023a [44]	Overall	182	--	15 (16.3) ^a^	--	--	108 (59.3)	36 (19.8)	--	77 (42.3)	--
Banerjee 2023b [25,26]	Overall	247	--	48 (19.4)	--	--	126 (51.0)	55 (22.3)	--	100 (40.5)	--
Dima 2023 [12,27,28,29,30]	Overall	106	88 (83)	56 (53)	35 (33)	33 (31)	27 (25)	2 (2)	21 (20)	14 (13)	14 (13)
	>70 years old	33	--	--	15 (45.0)	--	--	--	--	--	--
	Patients with EMD	45	--	25 (55.5)	16 (35.5)	--	--	--	--	--	--
Faiman 2023 [63]	Overall	26	--	9 (38)	--	--	--	--	--	--	--
Firestone 2023 [17,31,32,33]	Overall	52	--	27 (52)	--	--	--	--	--	--	--
Ghamsari 2024 [64]	Overall	18	--	7 (39)	--	--	--	--	--	--	--
Gordon 2023 [66]	Overall	58	51 (87.9)	23 (39.7)	17 (29.8)	(48.3)	--	--	--	13 (22.4)	--
Graf 2024 [67]	Overall	25	--	11 (44)	--	--	--	--	--	--	--
Grajales-Cruz 2023 [68,69]	Overall	36	36 (100.0)	36 (100.0)	--	--	7 (22.6)	2 (6.45)	10 (32.3)	--	6 (18.8)
Howard 2023 [47]	Overall	23	--	8 (35)	--	--	--	--	--	--	--
Kowalski 2023 [42]	Overall ^c^	31	26 (84)	4 (13)	16 (52) ^d^	15 (48) ^e^	--	--	--	--	--
Lachenal 2023 [74]	Overall	15	--	--	--	--	--	--	--	15 (100) ^f^	--
Midha 2023 [22]	Overall	56	45 (80.0)	20 (35.7)	17 (30.4)	--	--	--	--	--	11 (19.6)
Mohan 2024 [19,34]	Overall	110	--	38 (35)	--	--	--	--	--	--	--
Perrot 2023 [75]	Overall	572	--	49 ^g^ (8.6)	108 ^g^ (18.9) ^d^	--	--	--	--	102 ^g^ (17.8)	--
Pianko 2024 [35]	Overall	419	--	102 (24.3)	--	--	164 (39.1)	50 (11.9)	--	206 (49.2)	--
Rees 2024 [76]	TEC treatment group ^b^	41	--	25 (61.0)	--	--	--	--	--	--	--
Riedhammer 2024 [20,36]	Overall	123	48 ^g^ (39)	45 (37.4)	--	--	--	--	--	--	--
Sandahl 2023 [7,77]	Overall	49	--	17 (34.7)	--	--	27 (55.1)	14 (28.6)	--	15 (30.6)	--
Tan-Asoori 2023 [37,38,39]	Overall	204	122 (70) ^h^	91 (45.0)	--	--	--	--	--	--	25 (12)
Tan 2023 [53,80]	Overall	113	--	--	--	--	65 (57.5)	--	--	32 (28.3)	--
Tan 2024 [40,41]	Overall	86	--	32 (37)	5 (10) ^i^	--	--	--	--	--	9 (10)
	Patients switched to less frequent dosing schedule	32	--	10 (31)	--	--	--	--	--	--	--

Notes: Double dashes (“—”) indicate data not reported. ^a^ Based on the evaluated population (n = 92); ^b^ overall population of combined multiple treatments, with TEC-only data limited; ^c^ the overall population was treated with prophylactic tocilizumab; ^d^ % hand calculated; ^e^ reported as hematological ineligibility; ^f^ reported as renal disease; ^g^ n hand calculated; ^h^ based on the evaluated population (n = 175); ^i^ based on the evaluable population n = 50. Abbreviations: BCMA, B-cell maturation antigen; CrCl, creatinine clearance; ECOG, Eastern Cooperative Oncology Group performance status; EMD, extramedullary disease; TCRT, T-cell redirection therapy; TCZ, tocilizumab, TEC, teclistamab.

**Table A10:** Summary of key effectiveness outcomes.

Study ID	Overall/Subgroup Details	Sample Size	Timepoint/mFU	Overall Response Rate, n (%)		
				PR or Better	VGPR or Better	CR or Better
Asoori 2023 [57]	Overall	46	Median of 3 months	32 ^b^ (70.0)	25 (54.3) ^c,d^	6 (13.0) ^c,d^
Bansal 2024 [59]	TEC treatment group ^a^	48	January 2024 cut-off	29 ^b^ (61.0)	--	--
Dima 2023 [12,27,28,29,30]	Overall	104	Median of 3.8 months	70 (66) ^b^	49 (46)^c^	31 (29) ^c^
	>70 years old	34	Median of 3.8 months ^e^	24 (71)	--	10 (30) ^f^
Faiman 2023 [63]	Overall	26	Median of 2.5 months	15 (60) ^c^	9 (36) ^c^	4 (16) ^c^
Firestone 2023 [17,31,32,33]	Overall	47	Median of 3.1 months	30 (64)	18 ^b^ (38)	--
	Prior anti-BCMA exposure	26	Median of 3.1 months ^e^	13 (50.0)	--	--
Faiman 2023 [63]	Overall	18	June 2023 cut-off	9 ^b^ (50)	9 ^b^ (50)	--
Gordon 2023 [66]	Overall	58	--	29 ^b^ (50.0)	14 ^b^ (24.1)	--
Grajales-Cruz 2023 [68,69]	Overall	36	Median of 4.2 months	19 ^b^ (52.8)	16 ^b^ (44.5) ^c^	16 ^b^ (44.5) ^c^
Hebraud 2023 [51]	Overall	8	July 2023 cut-off	--	6 (75) ^d^	--
Kowalski 2023 [42]	Patients with secretory disease ^g^	30	Median of 3.4 months ^h^	15 (50)	--	9 (30) ^c^
Kumar 2023 [73]	Overall	9	3-month evaluation	6 (66.7)	4 (44.4) ^c^	3 (33.3) ^c^
Lachenal 2023 [74]	Overall	15	Median of 5.2 months	13 (86.7) ^d^	11 (73.3) ^c,e^	4 (26.7) ^c,d^
Midha 2023 [22]	Overall	56	Median of 2.3 months ^h^	30 ^b^ (53.6)	--	--
Mohan 2024 [19,34]	Overall	98	Median of 3.5 months	61 (62)	50 ^b^ (51)	20 ^b^ (20)
Nader 2023 [23]	Overall	27	Median of 33.6 months ^i^	19 (70)	--	--
Riedhammer 2024 [20,36]	Overall	123	Median of 5.5 months	73 ^b^ (59.3)	59 ^b^ (48.0) ^c^	27 ^b^ (22.0) ^c,j^
Schaefers 2023 [78]	Overall	16	Median of 3.4 months ^h^	7 ^b^ (44)	5 ^b^ (31)	--
Tan-Asoori 2023 [37,38,39]	Overall	180	Median of 5 months	115 ^b^ (64)	90 ^b^ (50) ^c^	34 ^b^ (19) ^c^
Tan 2024 [40,41]	Overall	77	Median of 9.5 months	47 (61)	33 (43)	--
	Prior BCMA-directed therapy	32	Median of 9.5 months ^e^	14 (43)	--	--
Venkatesh 2023 [82]	Overall	22	Median of 3.1 months	11 ^b^ (50)	--	--

Notes: Double dashes (“—”) indicate data not reported. ^a^ Overall population in which TEC was studied and other interventions in a combined cohort, the stratified subgroup data are presented here and are included in the overall ranges; ^b^ n hand calculated; ^c^ calculated from stratified best response; ^d^ based on July 2023 cut-off (n = 33); ^e^ the median follow-up was assumed to apply to the subgroup data, as both share the same data cut-off; ^f^ % hand calculated; ^g^ the population was treated with prophylactic tocilizumab; ^h^ converted from days to months; ^i^ converted from years to months; ^j^ reported as near complete or complete response. Abbreviations: BCMA, B-cell maturation antigen; CR, complete response; mFU, median follow-up; PR, partial response; TEC, teclistamab; VGPR, very good partial response.

**Table A11:** Summary of key safety outcomes.

Study ID	Overall/Subgroup Details	Sample Size	Timepoint/mFU	CRS, n (%)				ICANS, n (%)				Infections, n (%)	
				Any Grade	Grade 1	Grade 2	Grade 3+	Any Grade	Grade 1	Grade 2	Grade 3+	Any Grade	Grade 3+
Chart review—mixed inpatients and outpatient or not reported													
Asoori 2023 [57]	Overall	46	Median of 3 months	--	--	--	--	--	--	--	--	39 (84.8)	14 (35.8) ^a^
Bansal 2023 [45,58]	Overall ^b^	24	July 2023 cut-off	13 (54.2)	--	--	--	--	--	--	--	--	--
Catamero 2023 [61]	Overall	26	May 2023 cut-off	20 ^c^ (78)	--	--	--	--	--	--	--	--	--
	CRS patients	20	May 2023 cut-off	--	17 ^c^ (85)	3 ^c^ (15)	0 (0)	--	--	--	--	--	--
Glenn 2024 [65]	Overall	18	--	1 (5.6)	--	--	--	--	--	--	--	--	--
Gordon 2023 [66]	Overall	58	--	30 ^c^ (52)	--	--	--	6 ^c^ (11)	--	--	--	--	--
Grajales-Cruz 2023 [68,69]	Overall	36	Median of 4.2 months	21 (58.3)	15 (45.5)	4 (12.1)	0 (0)	--	--	--	--	21 (58.3)	--
Hamadeh 2024 [18,70,71]	Overall	72	July 2023 cut-off	46 ^c^ (63.9) ^d^	36 ^c^ (50.0) ^d^	10 ^c^ (13.9) ^d^	--	--	--	--	--	--	--
	Prior TCRT	27	July 2023 cut-off	10 ^c^ (37)	8 ^c^ (30)	2 ^c^ (7)	--	--	--	--	--	--	--
	No prior TCRT	45	July 2023 cut-off	36 ^c^ (80)	28 ^c^ (62)	8 ^c^ (18)	--	--	--	--	--	--	--
Kawasaki 2024 [72]	Overall	27	--	--	13 ^c^ (48.1) ^d,e^		--	4 ^c^ (14.8) ^d^	--	--	--	--	--
	Dosing schedule 1, 3, 5 (days)	23	--	--	11 (48) ^e^		--	4 (17)	--	--	--	--	--
	Dosing schedule 1, 4, 7 (days)	4	--	--	2 (50) ^e^		--	0 (0)	--	--	--	--	--
Kowalski 2023 [42]	Prophylactic tocilizumab	31	Median of 3.4 months ^f^	4 ^c^ (13)	--	--	--	3 ^c^ (10)	--	--	--	8 (26)	--
Kumar 2023 [73]	Overall	9	3-month evaluation	7 (77.8) ^d^	--	--	--	1 (11.1) ^d^	--	--	--	--	--
Midha 2023 [22]	Overall	56	Median of 2.3 months ^f^	29 (51.8)	--	--	1 (1.8)	--	--	--	--	32 (57.1)	--
Mohan 2024 [19,34]	Overall	110	Median of 3.5 months	62 ^c^ (56)	57 ^c^ (51.8) ^d,e^		5 (4.5) ^d^	12 (11)	--	--	5 (4.5)	44 (40)	29 (26)
Riedhammer 2024 [20,36]	Overall	123	Median of 5.5 months	72 (58.5)	--	--	2 (1.6)	--	--	--	--	67 (54.5)	33 (26.8)
Tabbara 2024 [79]	Overall	25	December 2023 cut-off	--	15 (60) ^e^		0 (0)	4 (16)	--	2 (8)	0 (0)	6 (24)	3 (12)
Tan-Asoori 2023 [37,38,39]	Overall	204	Median of 5 months	110 (53.9) ^c,d^	84 (41.2) ^c,d^	25 (12.3) ^c,d^	1 (0.5) ^c,d^	--	--	--	--	115 (60.0)	--
Venkatesh 2023 [82]	Overall	22	Median of 3.1 months	--	9 (41.0) ^d,e^		--	5 (23)	--	--	2 (9)	--	--
Chart review—inpatient monitoring													
Bolton 2024 [60]	Overall	9	--	--	3 (33.3)	--	--	--	2 (22.2)	--	--	--	--
Dima 2023 [12,27,28,29,30]	Overall	106	Median of 3.8 months	68 (64)	57 (54)	10 (9)	1 (1)	15 (14)	5 (5)	7 (6)	3 (3)	33 (31)	--
	>70 years old	33	July 2023 cut-off	22 (67)	--	--	1 (3)	7 (21)	--	--	0 (0)	11 (33)	--
Faiman 2023 [63]	Overall	26	Median of 2.5 months	22 (85)	--	--	--	5 (19)	--	--	--	7 (26.9) ^e^	--
Firestone 2023 [17,31,32,33]	Overall	52	Median of 3.1 months	27 (52)	--	--	--	--	--	--	--	--	--
Ghamsari 2024 [64]	Overall	18	June 2023 cut-off	12 (67)	--	4 (22.2) ^d^	0 (0)	2 (11)	--	1 (5.6) ^d^	--	12 ^c^ (67)	--
Graf 2024 [67]	Overall	25	August 2023 cut-off	13 (52)	12 (48)	1 (4)	--	1 (4)	--	--	--	--	--
Marin 2023 [43]	Overall	53	August 2023 cut-off	21 ^c^ (39.6) ^g^	--	--	--	5 (9.4) ^c^	--	--	--	--	--
	Prophylactic TCZ	38	August 2023 cut-off	10 ^c^ (26.3)	--	--	--	2 ^c^ (5.3)	--	--	--	--	--
	No prophylactic TCZ	15	August 2023 cut-off	11 ^c^ (73.3)	--	--	--	3 ^c^ (20.0)	--	--	--	--	--
Mooney 2024 [46]	Patients admitted to hospital	15	--	13 (86.7) ^d,h^	--	--	--	13 (86.7) ^d,h^	--	--	--	--	--
Schaefers 2023 [78]	Overall	16	Median of 3.4 months ^f^	4 (25)	--	--	0 (0)	0 (0)	--	--	0 (0)	13 (81)	9 (56)
Tan-Asoori 2023 [37,38,39]	Inpatients	160	Median of 5 months	94 (59)	72 (45)	22 (14)	0 (0)	--	--	--	--	--	--
Chart review—outpatient monitoring													
Hebraud 2023 [51]	Overall	8	July 2023 cut-off	3 (37.5) ^d^	--	--	--	--	--	--	--	--	--
Sandahl 2023 [7,77]	Overall	45	September 2023 cut-off	13 (28.9)	10 (22.2)	2 (4.4)	1 (2.2)	2 (4.4)	--	--	--	--	--
Tan-Asoori 2023 [37,38,39]	Outpatients	44	Median of 5 months	16 (36)	12 (27)	3 (7)	1 (2)	--	--	--	--	--	--
Varshavsky-Yanovsky 2023 [9,81]	Overall	18	6-month evaluation	--	5 (27.8)	1 (5.6)	--	--	--	--	--	--	--
Secondary databases													
Banerjee 2023a [44]	Overall	131	July 2023 cut-off	54 (41.2)	39 (29.8)	9 (6.9)	2 (1.5)	--	--	--	--	--	--
Banerjee 2023b [25,26]	Overall	76	Median of 5.1 months	14 (18.4)	8 (10.5)	3 (3.9)	1 (1.3)	3 (3.9)	2 (2.6)	1 (1.3)	0 (0)	--	--
Gong 2023 [21]	Total count of AEs	1317 events ^i^	--	130 (9.5) events	--	--	--	34 (2.5) events	--	--	--	214 (15.7) events	--
Lachenal 2023 [74]	Overall	15	Median of 5.2 months	--	11 (73.3) ^d,e^		--	1 (6.7) ^d^	--	--	--	9 (60.0) ^d^	--
Tan 2023 [53,80]	Overall	58	April 2023 cut-off	15 (25.9)	11 (19.0)	4 (6.9)	--	--	--	--	--	--	--

Notes: Double dashes (“—”) indicate data not reported. Results are based on the most recent timepoint in each study. ^a^ Reported as grade ¾ infections; ^b^ the most recent data cut-off no longer reports TEC alone and is not included in the overall population summary ranges; ^c^ n hand calculated; ^d^ % hand calculated; ^e^ reported as grade 1 and 2; ^f^ converted from days to months; ^g^ only the no tocilizumab cohort of the overall population is included in the ranges; ^h^ reported as CRS and/or ICANS; ^i^ total number of events—note that this differs from other studies which report the total number of patients. Abbreviations: AE, adverse event; CRS, cytokine release syndrome; ICANS, immune effector cell-associated neurotoxicity syndrome; TCRT, T-cell redirection therapies; TCZ, tocilizumab.

## Appendix F. Additional Patient Characteristics and Effectiveness Outcomes

**Table A12:** Summary of prior lines of therapy.

Study ID	Overall/Subgroup Details	n	Prior BCMA CAR T, n (%)	Prior BCMA ADC, n (%)	Prior BCMA BsAb, n (%)	Prior Non-BCMA, n (%)		Prior AlloSCT, n (%)	Prior AutoSCT, n (%)	Triple-Class, n (%)		Penta-Class, n (%)	
						CAR-T	BsAb			Exposed	Refractory	Exposed	Refractory
Asoori 2023 [57]	Overall	46	9 (19.6) ^a^	4 (8.7) ^a^	3 (6.5) ^a^	--	--	--	--	--	41 (89.1)	--	--
Banerjee 2023a [44]	Overall	92	8 (8.7)	9 (9.8) ^b^	--	--	--	43 (46.7)		--	--	--	--
Banerjee 2023b [25,26]	Overall	247	27 (10.9)	25 (10.1)	1 (<1)	--	--	--	--	--	--	--	--
Bansal 2024 [59]	TEC treatment group	48	20 (71.4)	--	--	--	--	--	--	--	--	--	--
Dima 2023 [12,27,28,29,30]	Overall	106	--	--	--	--	--	3 (3)	61 (58)	--	97 (92)	--	68 (64)
	>70 years old	33	--	--	--	--	--	--	19 (58)	--	32 (97)	--	19 (58)
	Patients with EMD	45	--	--	--	--	--	--	29 (64)	--	42 (93)	--	26 (58)
Faiman 2023 [63]	Overall	26	--	1 (3.8) ^a^	--	--	--	10 (38) ^c^		--	26 (100)	--	(92)
Firestone 2023 [17,31,32,33]	Overall	52	19 (37)	16 (31)	2 (4)	3 (6)	5 (10)	3 (6)	40 (77)	50 (96)	--	--	35 (67)
Ghamsari 2024 [64]	Overall	18	5 (27.8) ^a^	2 (11.1) ^a^	--	--	--	--	--	--	18 (100.0)	--	17 (94.4)
Gordon 2023 [66]	Overall	58	12 (20.7)	15 (25.9) ^b^	--	--	--	2 (3.4)	40 (69)	58 (100.0)	--	42 (72.4)	--
Graf 2024 [67]	Overall	25	--	--	--	---	--	--	--	--	20 (80)	--	12 (48)
Grajales-Cruz 2023 [68,69]	Overall	36	26 (72.2)	4 (11.1)	--	--	--	--	25/33 (75.8) ^c^	--	35 (97.2)	--	22 (61.1)
Hamadeh 2024 [18,70,71]	Overall	72	19 (27.5) ^d^	--	3 (4.2) ^d^	2 (2.8) ^d,e^	7 (9.7) ^d,f^	5 (6.9) ^d^	48 (66.7) ^d^	--	--	--	--
	Prior TCRT	27	19/21 (90)	--	3/10 (30)	2/21 (10) ^e^	7/10 (70) ^f^	3 (11)	19 (70)	--	--	--	--
	No prior TCRT	45	--	--	--	--	--	2 (1)	29 (64)	--	--	--	--
Howard 2023 [47]	Overall	23	--	--	--	--	--	1 (4)	16 (70)	--	20 (87)	--	10 (43)
Kawasaki 2024 [72]	Overall	27	--	--	--	--	--	--	18 (66.7)	--	27 (100.0) ^d^	--	--
	Dosing schedule 1, 3, 5 (days)	23	--	--	--	--	--	--	15 (65)	--	23 (100)	--	--
	Dosing schedule 1, 4, 7 (days)	4	--	--	--	--	--	--	3 (75)	--	4 (100)	--	--
Kowalski 2023 [42]	Overall ^g^	31	--	--	--	--	--	12 (39) ^h^		31 (100)	26 (84)	21 (68)	6 (19)
Marin 2023 [43]	Overall	53	--	--	--	--	--	--	--	--	53 (100.0) ^d,i^	--	--
	No prophylactic TCZ	15	--	--	--	--	--	--	--	--	15 (100.0) ^i^	--	--
	Prophylactic TCZ	38	--	--	--	--	--	--	--	--	38 (100.0) ^i^	--	--
Midha 2023 [22]	Overall	56	13 (23.2)	12 (23.2)	--	--	--	33 (58.9) ^j^		--	--	--	--
Mohan 2024 [19,34]	Overall	110	--	--	--	--	--	--	96 (87)	--	95 (86)	--	84 (76)
Perrot 2023 [75]	Overall	572	--	--	--	--	--	--	--	--	398 ^k^ (69.6)	--	183 ^k^ (32.0)
Pianko 2024 [35]	Overall	419	51 (12.2)	71 (16.9)	0 (0.0)	--	--	--	--	--	--	--	--
Riedhammer 2024 [20,36]	Overall	123	21 (17.1) ^a,l^	23 (18.7) ^a^	--	--	--	--	--	--	114 ^k^ (92.6)	--	74 ^k^ (60.2)
Sandahl 2023 [7,77]	Overall	49	10 (20.4)	5 (10.2)	--	--	--	--	--	--	--	--	--
Schaefers 2023 [78]	Overall	16	--	--	--	--	--	--	--	--	--	--	16 (100)
Tabbara 2024 [79]	Overall	25	1 (4)	--	--	--	--	13 (52) ^j^		--	--	--	--
Tan-Asoori 2023 [37,38,39]	Overall	204	40 (19.6) ^a^	25 (12.3) ^a^	5 (2.5) ^a^	--	--	--	--	--	123/156 (79.0)	--	56/150 (37.0)
Tan 2024 [40,41]	Overall	86	21/32 (66)	19/32 (60)	3/32 (9)	--	--	3 (3)	53 (62)	--	--	--	--
Venkatesh 2023 [82]	Overall	22	--	--	--	--	--	17 (77) ^j^		--	20 (91)	--	13 (59)

Notes: Double dashes (“—”) indicate data not reported. The table only includes studies where the relevant characteristics were reported. ^a^ % hand calculated; ^b^ reported as belantamab; ^c^ reported as HSCT; ^d^ calculated from stratified cohorts; ^e^ includes one (5%) GPRC5D and one (5%) CD38-targeted CAR-T therapy; ^f^ includes three (30%) GPRC5D and four (40%) FcRH5-targeted BsAB therapies; ^g^ the overall population was treated with prophylactic tocilizumab; ^h^ reported as SCT; ^i^ IMID, PI, and CD38 mAb refractory; ^j^ allogenic or autologous not distinguished; ^k^ n hand calculated; ^l^ reported as ide-cel. Abbreviations: ADC, antibody-drug conjugate; AlloSCT, allogenic stem cell transplant; AutoSCT, autologous stem cell transplant; BCMA, B-cell maturation antigen; BsAB, bispecific antibody; CAR-T, chimeric antigen receptor t-cell; CD-38, cluster of differentiation 38; EMD, extramedullary disease; GPRC5D, G protein-coupled receptor, class C, group 5, member D; IMID, immunomodulatory imide drugs; HSCT, hemopoietic stem cell therapy; mAb, monoclonal antibody; PI, proteasome inhibitors; SCT, stem cell therapy; TCRT, T-cell redirection; TCZ, tocilizumab; TEC, teclistamab.

**Table A13:** Summary of comorbidities at baseline.

Study ID	Overall/Subgroup Details	Sample Size	Hemodialysis/dialysis, n (%)	Peripheral Neuropathy, n (%)	Hypogammaglobulinemia, n (%)	CNS Involvement, n (%)
Asoori 2023 [57]	Overall	46	2 (5.4)	--	32 (69.6)	--
Banerjee 2023a [44]	Overall	124	9 (4.9)	57 (31.3)	52 (28.6)	--
Banerjee 2023b [25,26]	Overall	247	--	89 (36)	39 (15.8)	--
Dima 2023 [12,27,28,29,30]	Overall	106	--	--	--	4 (4)
Grajales-Cruz 2023 [68,69]	Overall	33	--	--	17 (53.1)	--
Kowalski 2023 [42]	Overall ^b^	31	--	--	--	2 (7)
Lachenal 2023 [74]	Overall	15	15 (100) ^c^	--	--	--
Midha 2023 [22]	Overall	56	--	--	--	7 (12.5)
Pianko 2024 [35]	Overall	419	--	--	125 (29.8)	--
Sandahl 2023 [7,78]	Overall	49	--	28 (57.1)	20 (40.8)	--
Tabbara 2024 [79]	Overall	25	1 (4.0)	--	--	--
Tan-Asoori 2023 [37,38,39]	Overall	204	2 (1) ^a^	--	105/153 (69)	--
Tan 2023 [53,80]	Overall	113	--	41 (36.3)	--	--
Tan 2024 [40,41]	Overall	86	2 (2)	35 (41)	--	--

Notes: Double dashes (“—”) indicate data not reported. The table only includes studies where the relevant characteristics were reported. ^a^ n hand calculated; ^b^ the overall population was treated with prophylactic tocilizumab; ^c^ assumed based on inclusion criteria. Abbreviations: CrCl, creatine clearance; CNS, central nervous system; TCRT, T-cell redirection therapy; TCZ, tocilizumab; TEC, teclistamab.

**Table A14:** Summary of key effectiveness outcomes stratified by best response.

Study ID	Overall/Subgroup Details	Sample Size	Timepoint/mFU	Overall Response Rate, n (%)			Stratified Best Response, n (%)					
				PR or Better	VGPR or Better	CR or Better	PD	SD	PR	VGPR	CR	sCR
Asoori 2023 [57]	Overall	46	Median of 3 months	32 ^a^ (70.0)	--	--	--	--	7 (15.2) ^b^	19 (41.3) ^b^	6 (13.0) ^b^	
Dima 2023 [12,27,28,29,30]	Overall	104	Median of 3.8 months	70 (66)	--	--	26 (24)	10 (9.5)	21 (20)	18 (17)	31 (29)	
	>70 years old	34	Median of 3.8 months	24 (71)	--	10 (30) ^c^	--	--	--	--	--	--
Faiman 2023 [63]	Overall	26	Median of 2.5 months	--	--	--	6 (24)	4 (16)	6 (24)	5 (20)	4 (16)	--
Firestone 2023 [17,31,32,33]	Overall	47	Median of 3.1 months	30 (64)	18 ^a^ (38)	--	--	--	--	--	--	--
Ghamsari 2024 [64]	Overall	18	June 2023 cut-off	9 ^a^ (50)	9 ^a^ (50)	--	--	--	--	--	--	--
Gordon 2023 [66]	Overall	58	--	29 ^a^ (50.0)	14 ^a^ (24.1)	--	--	8 ^a^ (13.1) ^b^	15 ^a^ (25.9)	14 ^a^ (24.1)	--	--
Grajales-Cruz 2023 [65,66]	Overall	36	Median of 4.2 months	19 ^a^ (52.8)	--	--	--	--	3 ^a^ (8.3)	0 (0.0)	16 ^a^ (44.5)	
Hebraud 2023 [51]	Overall	8	July 2023 cut-off	--	6 (75) ^b^	--	--	--	--	--	--	--
Kowalski 2023 [42]	Patients with secretory disease ^d^	30	Median of 3.4 months ^e^	15 (50)	--	--	4 (13)	11 (37)	4 (13)	--	9 (30)	--
Kumar 2023 [73]	Overall	9	3 months	6 (66.7)	--	--	3 (33.3) ^b^	--	2 (22.2) ^b^	1 (11.1) ^b^	1 (11.1) ^b^	2 (22.2) ^b^
Lachenal 2023 [74]	Overall	15	Median of 5.2 months	13 (86.7) ^b^	--	--	--	--	2 (13.3) ^b^	7 (46.7) ^b^	2 (13.3) ^b^	2 (13.3) ^b^
Midha 2023 [22]	Overall	56	Median of 2.3 months ^e^	30 ^a^ (53.6)	--	--	--	--	--	--	--	--
Mohan 2024 [19,34]	Overall	98	Median of 3.5 months	61 (62)	50 ^a^ (51)	20 ^a^ (20)	--	--	--	--	--	--
Nader 2023 [23]	Overall	27	33.6 months ^f^	19 (70)	--	--	--	--	--	--	--	--
Riedhammer 2024 [20,36]	Overall	123	Median of 5.5 months	73 ^a^ (59.3)	--	--	--	--	14 ^a^ (11.4)	32 ^a^ (26.0)	27 ^a^ (22.0) ^g^	
Schaefers 2023 [78]	Overall	16	3.4 months ^e^	7 ^a^ (44)	5 ^a^ (31)	--	7 ^a^ (44)	2 ^a^ (13)	2 ^a^ (13)	--	--	--
Tan-Asoori 2023 [37,38,39]	Overall	180	Median of 5 months	115 ^a^ (64)	--	--	40 ^a^ (22)	25 ^a^ (14)	25 ^a^ (14)	56 ^a^ (31)	34 ^a^ (19)	
Tan 2024 [40,41]	Overall	86	Median of 9.5 months	47 (61.0)	33 (43.0)	--	--	--	--	--	--	--
	Prior BCMA-directed therapy	32	Median of 9.5 months	14 (43)	--	--	--	--	--	--	--	--
Venkatesh 2023 [82]	Overall	22	Median of 3.1 months	11 ^a^ (50)	--	--	--	--	--	--	--	

Notes: Double dashes (“—”) indicate data not reported. The table only includes studies where the relevant outcomes were reported. ^a^ n hand calculated; ^b^ % hand calculated; ^c^ based on July 2023 cut-off (n = 33); ^d^ the population was treated with prophylactic tocilizumab; ^e^ converted from days to months; ^f^ converted from years to months; ^g^ reported as near complete or complete response. Abbreviations: BCMA, B-cell maturation antigen; CI, confidence interval; CR, complete response; NA, not available; NR, not reached; PD, progression of disease; PR, partial response; sCR, stringent complete response; SD, stable disease; TEC, teclistamab; VGPR, very good partial response.

## Appendix G. Summary of MajesTEC-1 Trial Characteristics and Outcomes

**Table A15:** Summary of MajesTEC-1 trial characteristics and outcomes.

Characteristic	MajesTEC-1 (n = 165)
Study description	
Study design	Clinical trial
MajesTEC-1 ineligibility, %	0
Median follow-up, months	23
Median prior LOTs	5
Select efficacy outcomes	
ORR, % (overall)	63
CR or better, %	45.5
VGPR or better, %	59.4
Median PFS, months (95% CI)	11.3 (8.8–16.4)
Median OS, months (95% CI)	21.9 (15.1—NE)
Select safety outcomes	
CRS (any grade), %	72
ICANS (any grade), %	3
Infections (any grade), %	80

Abbreviations: CR, complete response; CRS, cytokine release syndrome; ICANS, immune effector cell-associated neurotoxicity syndrome; LOT, line of therapy; NE, not estimated; ORR, overall response rate; OS, overall survival; PFS, progression-free survival.
