# Supplementary material for: Real-World Evidence Evaluating Teclistamab in Patients with Relapsed/Refractory Multiple Myeloma: A Systematic Literature Review
Source: Cancers (Basel). 2025 Apr 5;17(7):1235. doi: 10.3390/cancers17071235 (PMC11988155; doi:10.3390/cancers17071235)
Supplement: Supplementary file 1 [file cancers-17-01235-s001.zip › REDACTED_TEC-3450 Systematic Literature Review-FD-JNJ-64007957-AAA-1554146.pdf]

PRECISION AQ™

A systematic literature review of real-world evidence evaluating teclistamab in patients with relapsed or refractory multiple myeloma

Systematic literature review protocol

Prepared for:

Janssen

PPD [redacted]

June 19, 2024

Version 4

This document contains proprietary and confidential information, ideas, and methodologies of Precision AQ, LLC, and shall not be used, disclosed or reproduced, in whole or in part, for any purpose other than for its intended purpose without the prior written consent of Precision AQ, LLC.

Study team

Study Sponsor: Johnson & Johnson Innovative Medicine

Project Lead: PPD

Project Team:

Precision team members:

PPD

Johnson & Johnson team members:

PPD

Version history

Version	Changes	Date issued
1	--	October 31, 2023
2	Incorporated feedback from Janssen	November 15, 2023
3	Incorporated an additional variable for data extraction and made minor revisions to the search strategies	November 24, 2023
4	Search strategies updated to incorporate revised number of hits for the first SLR update; minor revisions to methods to include additional conference screening and clarification of variables for extraction	June 19, 2024

Copyright 2024 Precision AQ, LLC

Contents

List of tables **5**

Abbreviations **6**

1 Introduction **8**

2 Objective **10**

3 Methodology **11**

3.1 Eligibility criteria 11

3.2 Study identification 12

3.3 Study selection 13

3.4 Data extraction 13

3.4.1 Study characteristics 13

3.4.2 Treatment characteristics 14

3.4.3 Patient characteristics 14

3.4.4 Outcomes 16

3.4.5 Risk of bias assessment 17

Appendix A – Literature search strategies **18**

Appendix B – Risk of bias assessment **21**

References **22**

Copyright 2024 Precision AQ, LLC

List of tables

Table 1: Eligibility criteria for systematic literature review 11

Table A.1: Search strategy for Embase 18

Table A.2: Search strategy for MEDLINE 19

Table A.3: Search strategy for Northern Light Life Sciences Conference Abstracts 20

Table B.1: Newcastle-Ottawa quality assessment scale – cohort studies 21

Abbreviations

ADC	Antibody-drug conjugate
AE	Adverse event
AMCP	Academy of Managed Care Pharmacy
ASCO	American Society of Clinical Oncology
ASH	American Society of Hematology
ASTCT	American Society for Transplantation and Cellular Therapy
BCMA	B-cell maturation antigen
CAR-T	Chimeric antigen receptor T-cell
CIBMTR	Center for International Blood and Marrow Transplant Research
CNS	Central nervous system
CrCl	Creatinine clearance
CRS	Cytokine release syndrome
ECOG	Eastern Cooperative Oncology Group
EHA	European Hematology Association
Embase	Excerpta Medica database
EMN	European Myeloma Network
HOPA	Hematology/Oncology Pharmacy Association
ICANS	Immune effector cell-associated neurotoxicity syndrome
ICU	Intensive care unit
IMS	International Myeloma Society
ISS	International Staging System
JADPRO	Journal of the Advanced Practitioner in Oncology
LL&M	Lymphoma, Leukemia & Myeloma
MEDLINE	Medical Literature Analysis and Retrieval System Online
MM	Multiple myeloma
NOS	Newcastle-Ottawa Scale
ONS	Oncology Nursing Society
PICOS	Population, intervention, comparator, outcome, and study design
PRISMA	Preferred Reporting Items for Systematic Reviews and Meta-Analyses
R-ISS	Revised International Staging System
RRMM	Relapsed or refractory multiple myeloma
SIGN	Scottish Intercollegiate Guidelines Network
SLR	Systematic literature review
SOHO	Society of Hematologic Oncology

Copyright 2024 Precision AQ, LLC

WHO	World Health Organization

Copyright 2024 Precision AQ, LLC

1 Introduction

Multiple myeloma (MM) accounts for 1.8% of all cancer diagnoses and 2.1% of deaths in the United States.[1] While 5-year survival rates of MM have more than doubled since the 1970s, they remain low at 52.9% among those with systemic disease, which constitute the vast majority of cases.[1] MM imposes substantial healthcare burden as patients progress through multiple lines of therapy; a recent retrospective database study found that all cause-related healthcare costs averaged $35,760 per patient per month among patients with four or more prior lines of therapy.[2] The disease is considered an incurable malignancy, and therefore, patients inevitably relapse and require multiple lines of therapy.[3] Treatment for relapsed or refractory multiple myeloma (RRMM) relies on providers considering factors such as timing of treatment for RRMM, patient comorbidities, and potential adverse events (AEs).[4] Current treatments for RRMM include proteasome inhibitors, immunomodulatory drugs, and anti-CD38 antibodies. The treatment landscape for RRMM has evolved considerably with the introduction of innovative targeted therapies, including B-cell maturation antigen (BCMA)-targeted chimeric antigen receptor T-cell and bispecific therapies.[5]

Approved in October 2022, Janssen's TECVAYLI™ (teclistamab-cqyv) is a first-in-class BCMA-targeted bispecific antibody for adult patients with RRMM who have previously received four or more (4+) prior lines of therapy, including a proteasome inhibitor, immunomodulatory drug, and anti-CD38 monoclonal antibody. Teclistamab is administered subcutaneously using a step-up dosing schedule given the risk of certain AEs (e.g., cytokine release syndrome [CRS] and neurotoxicity) and like many other cancer therapies, it is also associated with an increased risk of infection. Patients should be hospitalized for 48 hours after receiving teclistamab. In addition to monitoring for and treating AEs, providers must also consider the transition of care for patients to continue teclistamab care in an outpatient or community setting if a patient was referred to receive teclistamab step-up dosing originally in an inpatient setting.

In 2023, Janssen, in partnership with Precision, conducted a qualitative research study comprising interviews with oncologists (predominantly based in academic practice settings) who were early adopters of teclistamab in treating RRMM. The qualitative data collection was designed to explore strategies and procedures established by these early adopters and to provide real-world evidence-based experiences to help inform other providers seeking real-world practice experience in the use of teclistamab (*TecPIONEER 1.0: Panel Interview of ONcology practice with Emergent Experience of teclistamab in the Real-world*). As teclistamab approached the one-year anniversary of commercial availability and is increasingly utilized by community practitioners, there is a unique opportunity to capture and describe the evolving adoption of teclistamab as well as associated step-up dosing practices and processes, transition of care, patient management strategies and patient care models among community providers in the United States.

As such, the TecPIONEER team will conduct a follow-on multi-phase mixed-methods study (*TecPIONEER 2.0*) with parallel workstreams comprising data collection through a systematic literature review (SLR), a

Copyright 2024 Precision AQ, LLC

quantitative survey, and qualitative interviews primarily focused on community-based providers who have experience with teclistamab and hospital-based providers whose teclistamab care models have evolved since TecPIONEER 1.0. After data collection and analyses has been completed for all three workstreams, a roundtable discussion will be held virtually or at an upcoming conference to validate and discuss the study findings and clinical implications, from which dissemination products including a manuscript and other deliverables will be developed.

PRECISION AQ™

2 Objective

The overall objective of TecPIONEER 2.0 is to generate rapid and in-depth real-world evidence on the latest practices in treating patients with teclistamab, including the care model, transition of care and AE management. The results aim to optimize adoption of and treatment outcomes associated with teclistamab in the academic and community settings, and to maximize the impact and relevance of teclistamab real-world evidence.

As part of TecPIONEER 2.0 to help meet the project objective, an SLR was performed to gather existing real-world evidence of teclistamab in adult patients with RRMM available up to and including data presented at the 2023 American Society of Hematology (ASH) Annual Meeting. To ensure this evidence base is kept up to date, the SLR will be updated to capture any relevant evidence published in 2024. The goal is the summarize the latest real-world outcomes of teclistamab, including patient profiles, effectiveness, safety, healthcare resource utilization and dosing schedule. The results will be used to support ongoing discussions with healthcare providers and dissemination products including manuscripts.

Copyright 2024 Precision AQ, LLC

3 Methodology

3.1 Eligibility criteria

Study eligibility criteria are defined in terms of the population, intervention, comparator, outcome, and study design (PICOS) structure outlined in **Table 1**, which will guide the identification and selection of studies for the SLR.

The target population includes adult patients (≥18 years) with MM. The only intervention of interest is teclistamab, but no restrictions will be applied to comparators. No restrictions will be applied to outcomes to ensure all relevant evidence is captured. Real-world observational studies (prospective and retrospective) will be included, while clinical trials, pooled analyses of clinical trials, case reports, case series, and narrative reviews will be excluded. Systematic literature reviews will be excluded, but the bibliographies of relevant SLRs will be reviewed to capture relevant citations. Only English-language publications of full-text articles and conference materials will be included.

The original SLR captured all citations published in 2023. Two updates to the SLR will be conducted: the first will capture citations up to the end of May 2024, and the second will capture citations up to the end of December 2024.

Table 1: Eligibility criteria for systematic literature review

Criteria	Inclusion	Exclusion
**Population**	• Adult (≥18 years) patients with multiple myeloma	• Patients aged <18 years
**Interventions**	• Teclistamab	• Interventions not listed
**Comparators**	• No restrictions	--
**Outcomes**	• No restrictions	--
**Study design**	• Real-world observational studies (prospective, retrospective)	• Clinical trials<br>• Case reports or case series<br>• Pooled analyses of trials<br>• Systematic literature reviews[a]<br>• Non-systematic/narrative reviews
**Publication type**	• Full text publications<br>• Conference abstracts/posters[b]	• Letters to editors<br>• Editorials<br>• Commentary<br>• Expert opinion<br>• Guidelines
**Language**	• Only studies published in English	• Studies published in a language other than English (even if abstract is in English)
**Times**	• Published between 2023 and 2024	• Published before 2023

***Notes: a)** Systematic reviews will be excluded, but bibliography of relevant systematic reviews will be reviewed to capture relevant citations; **b)** Conference abstract/poster citations captured through search of Embase will be screened, and conferences of interest will also be searched separately in the Northern Light database or published proceedings from target conferences.*

Copyright 2024 Precision AQ, LLC

3.2 Study identification

Relevant studies will be identified by searching the following databases through the Ovid platform: Medical Literature Analysis and Retrieval System Online (MEDLINE) and Excerpta Medica database (Embase). The specific search algorithms will include a combination of indexing and free-text terms (see search terms in **Appendix A**; note, the same search strategies used for the original SLR will be re-run for both SLR updates). The population terms will be adapted from existing reviews,[6,7] and terms for the generic and brand names of teclistamab will be incorporated. The study design filters recommended by the Scottish Intercollegiate Guidelines Network (SIGN) for observational studies will be used to inform the search strategy (https://www.sign.ac.uk/what-we-do/methodology/search-filters/).[8] Database searches will be restricted to English language publications, with a date restriction from January 1, 2023 onwards.

The main database searches will be augmented with searches of specific conference proceedings. The Northern Light database will be used to search for studies from conferences that are indexed in the database, while conference websites will be hand searched for the remaining conferences. Posters or slides corresponding to the conference abstracts will be identified where available. Of note, some of the conferences will take place outside of the search dates, so citation screening will be performed for these conferences once the materials become available (e.g. ASH Annual Meeting). The following conferences will be searched:

- Academy of Managed Care Pharmacy (AMCP) Meeting
- AMCP Nexus[i]
- American Society for Transplantation and Cellular Therapy (ASTCT) and Center for International Blood and Marrow Transplant Research (CIBMTR) Tandem Meeting[i]
- American Society of Clinical Oncology (ASCO) Annual Meeting
- American Society of Hematology (ASH) Annual Meeting
- European Hematology Association (EHA) Annual Congress
- European Society for Medical Oncology (ESMO)
- Hematology/Oncology Pharmacy Association (HOPA) Annual Conference[i]
- International Myeloma Society (IMS) Annual Meeting
- International Conference on Oncology and Research Treatment
- Journal of the Advanced Practitioner in Oncology (JADPRO) Live[i]
- Lymphoma, Leukemia & Myeloma (LL&M) Congress[i]
- Oncology Nursing Society (ONS) Congress
- Society of Hematologic Oncology (SOHO) Annual Meeting

---

[i] Conference is not indexed in Northern Light database so the conference website will be hand searched.

Copyright 2024 Precision AQ, LLC

- European Myeloma Network (EMN) Meeting[i]

Any published and indexed conference abstracts that are not part of the above conferences will also be captured. Finally, the above searches will be supplemented with hand searches of the bibliographies of recent peer-reviewed systematic reviews that are identified through the database searches. These references will serve only as secondary sources to ensure that all key studies are identified.

3.3 Study selection

One reviewer will screen all titles, abstracts, and proceedings identified by the searches accordingly to the PICOS criteria. All citations identified as eligible during title/abstract screening will then be screened at a full-text stage by the same reviewer. The full-text studies identified at this stage will be included for data extraction. In each selection phase, a senior reviewer will quality-check/verify the inclusion/exclusion decisions and reconcile differences in case of disagreements. If necessary, a third reviewer will be included to reach consensus on any remaining discrepancies. Screening decisions will be stored and managed in a Microsoft Excel workbook. The process of study identification and selection will be summarized with a Preferred Reporting Items for Systematic Reviews and Meta-Analyses (PRISMA) flow diagram, including reasons for exclusion at the full-text stage.[9] Included publications will be mapped to studies so that each study is only counted once. The first full-text published will be considered the primary publication for a study and all others will be considered subsequent publications.

3.4 Data extraction

Data will be extracted from the final list of included studies. All data of interest will be extracted from primary publications, whereas only additional data reported for relevant outcomes or subgroups of interest will be extracted from subsequent publications. Data extraction will be conducted by one reviewer and quality checked by a senior reviewer. Any discrepancies between reviewers will be resolved through discussion, involving a third reviewer if necessary. Data will be stored and managed in a Microsoft Excel workbook. The study characteristics, treatment characteristics, patient characteristics, and outcomes that will be extracted are outlined below.

3.4.1 Study characteristics

The following study characteristics will be extracted:

- Study title
- Authors

Copyright 2024 Precision AQ, LLC

- Publication month/year
- Publication type (e.g., abstract, poster, slides, manuscript)
- Study design (e.g., retrospective observational study, prospective observational study)
- Study start and end dates/data timeframe
- Sample size
- Data sources (e.g., payer claims, electronic medical records, chart review)
- Study setting (e.g., academic centers, community-based practice, single center, multi-center, patient advocacy group)
- Geographical region (country/region of patient enrollment)
- Study objectives
- Study inclusion and exclusion criteria
- Treatment arms
- Study outcomes (primary, secondary, and exploratory), including definitions
- Pre-planned subgroups
- Length of follow-up period

3.4.2 Treatment characteristics

The following treatment characteristics will be extracted:

- Treatment regimen
- Treatment dose
- Method of administration
- Frequency of administration
- Treatment duration
- Concomitant/background therapies
- Pre-medications
- Healthcare setting for step-up dosing (e.g., inpatient, outpatient)

3.4.3 Patient characteristics

The following patient characteristics will be extracted:

- Age (including mean age, proportion of patients ≥75 years of age)
- Sex
- Race
- Ethnicity

Copyright 2024 Precision AQ, LLC

- Time since diagnosis
- Performance status (e.g., Eastern Cooperative Oncology Group [ECOG], World Health Organization [WHO], Karnofsky)
- International Staging System (ISS)/Revised International Staging System (R-ISS) stage
- Time since diagnosis
- Prior treatment experience
  - Number of prior lines of therapy
  - Type of treatment, such as:
    - Anti-BCMA therapy, including BCMA chimeric antigen receptor T-cell (CAR-T) therapy (cilta-cel, ide-cel), BCMA antibody-drug conjugate (ADC) therapy (belantamab mafodotin), BCMA bispecific antibodies, and multiple prior anti-BCMA, if reported
    - Non-BCMA T-cell receptor therapy (CAR-T, bispecific antibodies)
    - Stem cell transplantation
    - Radiation therapy
  - Therapy refractoriness (e.g., triple-class exposed, penta-drug refractory)
- Baseline comorbidities
  - Comorbidity index score, if available
  - Creatinine clearance (CrCl) <30mL/min or 40mL/min
  - Renal impairment/failure
    - End stage renal disease
  - Hemodialysis/dialysis
  - Peripheral neuropathy
  - Hepatic impairment
  - Hypogammaglobulinemia
  - Extramedullary disease
    - Central nervous system (CNS) involvement
  - Cytopenia (neutropenia ANC <1000 cells/uL; anemia [hemoglobin <8g/dL]; thrombocytopenia [platelets <75K cells/uL])
- High-risk cytogenetics
- MajesTEC-1 ineligibility (proportion of patients not meeting MajesTEC-1 study eligibility)
  - Top 3 reasons for MajesTEC-1 ineligibility as reported by authors
- Payer type (e.g., Medicare, Medicaid, commercial, managed care, other)
- Subgroups (e.g., patients who are elderly and frail, patients who have renal impairment)

Copyright 2024 Precision AQ, LLC

3.4.4 Outcomes

The following outcomes of interest will be extracted for the overall population and any relevant subgroups:

- Efficacy outcomes (including proportion of patients with events, median, dispersion [e.g., 95% CI])
    - Overall survival
    - Progression-free survival
    - Measures of response, including overall response rate, best response, time to response, duration of response
- Safety outcomes (including proportion of patients with events, grade of events)
    - CRS
    - Immune effector cell-associated neurotoxicity syndrome (ICANS)
    - Non-ICANS neurotoxicity
    - Infections
    - Cytopenia (neutropenia, leukopenia, anemia, thrombocytopenia, lymphopenia)
    - Discontinuations due to AEs
    - Mortality
- Healthcare provider practices
    - Treatment patterns
    - Dosing schedule
    - Procedures and experiences with teclistamab adoption
    - Remote monitoring
    - Transition of care
    - Processes for monitoring and managing AEs (including use of tocilizumab and intravenous immune globulin as prophylactic or reactive treatment)
- Healthcare resource utilization (including proportion of patients with events, median, mean, dispersion)
    - Outpatient visits
    - Inpatient visits/hospital admissions/readmissions
    - Emergency room visits
    - Intensive care unit (ICU) visits
    - Length of stay
    - Unscheduled physician communications

Copyright 2024 Precision AQ, LLC

3.4.5 Risk of bias assessment

One reviewer will assess study quality, followed by verification by a senior reviewer. Study quality assessments will only be performed for studies where full-text publications are available. The Newcastle-Ottawa Scale (NOS) will be used to assess the quality of observational studies (**Appendix B**).[10] This instrument is used to evaluate the quality of studies based on 1) study group and selection, 2) comparability of the groups within studies, and 3) the ascertainment of either the exposure or outcomes of interest for case-control or cohort studies. Ranking of the study quality will be done by using a ‘star system’ in which a study can be given a maximum of one star for each numbered item within the “Selection” and “Exposure” categories and a maximum of two stars for “Comparability” category.

Copyright 2024 Precision AQ, LLC

PRECISION AQ™

Appendix A – Literature search strategies

Table A.1: Search strategy for Embase

Database: Embase 1974 to May 22, 2024<br>Search executed on May 23, 2024			
**#**	**Criteria**	**Search terms**	**Results**
1	Population terms	exp multiple myeloma/	102,883
2		exp plasmacytoma/	14,274
3		exp Paraproteinemias/	183,815
4		(myeloma or (multiple adj2 myeloma$) or plasmacytom$ or plasmocytom$ or mgus or (monoclonal adj2 gammopath$)).mp.	149,247
5		or/1-4	221,071
6	Intervention terms	exp teclistamab/	406
7		(teclistamab or Tecvayli or JNJ-64007957 or teclistamab-cqyv).mp.	424
8		or/6-7	424
9	SIGN filters for observational studies	Clinical study/	166,793
10		Case control study/	217,895
11		Family study/	25,824
12		Longitudinal study/	213,746
13		Retrospective study/	1,624,202
14		Prospective study/	919,580
15		Randomized controlled trials/	274,328
16		14 not 15	908,367
17		Cohort analysis/	1,167,616
18		(Cohort adj (study or studies)).mp.	517,217
19		(Case control adj (study or studies)).tw.	176,157
20		(follow up adj (study or studies)).tw.	76,223
21		(observational adj (study or studies)).tw.	276,600
22		(epidemiologic$ adj (study or studies)).tw.	125,861
23		(cross sectional adj (study or studies)).tw.	373,627
24		(retrospective adj7 (study or studies or design or analysis or analyses or cohort or data or review)).ti,ab.	1,254,000
25		or/9-13,16-24	4,466,453
26	Combined criteria	5 and 8 and 25	96
27	Language filter	limit 26 to english language	96
28	Date filter	limit 27 to yr="2023 -Current"	72

Copyright 2024 Precision AQ, LLC

Table A.2: Search strategy for MEDLINE

Database: Ovid MEDLINE(R) ALL <1946 to May 22, 2024> Search executed on May 23, 2024			
#	Criteria	Search terms	Results
1	Population terms	exp multiple myeloma/	49,135
2		exp plasmacytoma/	8,932
3		exp Paraproteinemias/	64,055
4		(myeloma or (multiple adj2 myeloma$) or plasmacytom$ or plasmocytom$ or mgus or (monoclonal adj2 gammopath$)).mp.	84,636
5		or/1-4	96,987
6	Intervention terms	(teclistamab or Tecvayli or JNJ-64007957 or teclistamab-cqyv).mp.	89
7	SIGN filters for observational studies	Epidemiologic studies/	9,543
8		exp case control studies/	1,506,567
9		exp cohort studies/	2,607,741
10		Case control.tw.	162,188
11		(cohort adj (study or studies)).tw.	351,806
12		Cohort analy$.tw.	13,035
13		(Follow up adj (study or studies)).tw.	58,204
14		(observational adj (study or studies)).tw.	178,369
15		Longitudinal.tw.	345,820
16		Retrospective.tw.	812,750
17		Cross sectional.tw.	562,700
18		Cross-sectional studies/	502,656
19		or/7-18	4,003,471
20	Combined criteria	5 and 6 and 19	11
21	Language filter	limit 20 to english language	11
22	Date filter	limit 21 to yr="2023 -Current"	11

Copyright 2024 Precision AQ, LLC

Table A.3: Search strategy for Northern Light Life Sciences Conference Abstracts

Database: Northern Light Life Sciences Conference Abstracts 2010 - 2024 Week 20<br>Search executed on May 23, 2024			
**#**	**Criteria**	**Search terms**	**Results**
1	Population terms	exp multiple myeloma/	27388
2		exp plasmacytoma/	1694
3		exp Paraproteinemias/	30424
4		(myeloma or (multiple adj2 myeloma$) or plasmacytom$ or plasmocytom$ or mgus or (monoclonal adj2 gammopath$)).mp.	30055
5		or/1-4	32556
6	Intervention terms	(teclistamab or Tecvayli or JNJ-64007957 or teclistamab-cqyv).mp.	102
7	Conference filter	Academy of Managed Care Pharmacy.cf.	2749
8		American Society of Clinical Oncology.cf.	79140
9		American Society of Hematology.cf.	66037
10		European Hematology Association.cf.	31727
11		European Society for Medical Oncology.cf.	22763
12		International Myeloma.cf.	2497
13		Oncology Nursing Society.cf.	1267
14		Society of Hematologic Oncology.cf.	2052
15		or/7-14	208232
16	Combined criteria	5 and 6 and 15	82
17	Date filter	limit 16 to yr="2023 -Current"	47

Copyright 2024 Precision AQ, LLC

Appendix B – Risk of bias assessment

Table B.1: Newcastle-Ottawa quality assessment scale – cohort studies

Domain	Response
Selection	
1. Representativeness of the exposed cohort	a. Truly representative of the average ________________ (describe) in the community*<br>b. Somewhat representative of the average _______________ in the community*<br>c. Selected group of users (e.g. nurses, volunteers)<br>d. No description of the derivation of the cohort
2. Selection of the non-exposed cohort	a. Drawn from the same community as the exposed cohort*<br>b. Drawn from a different source<br>c. No description of the derivation of the non-exposed cohort
3. Ascertainment of exposure	a. Secure record (e.g. surgical records)*<br>b. Structured interview*<br>c. Written self-report<br>d. No description
4. Demonstration that outcome of interest was not present at start of study	a. Yes*<br>b. No
Comparability	
1. Comparability of cohorts on the basis of the design or analysis	a. Study controls for ______________ (select the most important factor)*<br>b. Study controls for any additional factor (this criteria could be modified to indicate specific control for a second important factor)*
Outcomes	
1. Assessment of outcome	a. Independent blind assessment*<br>b. Record linkage*<br>c. Self-report<br>d. No description
2. Was follow-up long enough for outcomes to occur	a. Yes (select an adequate follow up period for outcome of interest)*<br>b. No
3. Adequacy of follow up of cohorts	a. Complete follow up - all subjects accounted for*<br>b. Subjects lost to follow up unlikely to introduce bias - small number lost - >____% (select an adequate %) follow up, or description provided of those lost)*<br>c. Follow up rate <____% (select an adequate %) and no description of those lost<br>d. No statement

Note: *A study can be awarded a maximum of one star for each numbered item within the selection and exposure categories. A maximum of two stars can be given for comparability.*

References

1. Padala SA, Barsouk A, Barsouk A, et al. Epidemiology, Staging, and Management of Multiple Myeloma. *Med Sci (Basel).* 2021;9(1).
2. Jagannath S, Joseph N, He J, et al. Healthcare Costs of Multiple Myeloma Patients with Four or More Prior Lines of Therapy, Including Triple-Class Exposure in the United States. *Oncol Ther.* 2022;10(2):411-420.
3. Hari P, Romanus D, Palumbo A, et al. Prolonged Duration of Therapy Is Associated With Improved Survival in Patients Treated for Relapsed/Refractory Multiple Myeloma in Routine Clinical Care in the United States. *Clin Lymphoma Myeloma Leuk.* 2018;18(2):152-160.
4. Bazarbachi AH, Al Hamed R, Malard F, Harousseau JL, Mohty M. Relapsed refractory multiple myeloma: a comprehensive overview. *Leukemia.* 2019;33(10):2343-2357.
5. Hernandez-Rivas JA, Rios-Tamayo R, Encinas C, Alonso R, Lahuerta JJ. The changing landscape of relapsed and/or refractory multiple myeloma (MM): fundamentals and controversies. *Biomark Res.* 2022;10(1):1.
6. Kumar A, Loughran T, Alsina M, Durie BG, Djulbegovic B. Management of multiple myeloma: a systematic review and critical appraisal of published studies. *Lancet Oncol.* 2003;4(5):293-304.
7. Maiese EM, Ainsworth C, Le Moine JG, Ahdesmaki O, Bell J, Hawe E. Comparative Efficacy of Treatments for Previously Treated Multiple Myeloma: A Systematic Literature Review and Network Meta-analysis. *Clin Ther.* 2018;40(3):480-494 e423.
8. Scottish Intercollegiate Guidelines Network; Health Improvement Scotland. SIGN Search Filters. 2021; https://www.sign.ac.uk/what-we-do/methodology/search-filters/.
9. Moher D, Liberati A, Tetzlaff J, Altman DG, Group P. Preferred reporting items for systematic reviews and meta-analyses: the PRISMA statement. *J Clin Epidemiol.* 2009;62(10):1006-1012.
10. Wells GS, O'Connell D, Peterson J, Welch V, Losos M. The Newcastle-Ottawa Scale (NOS) for assessing the quality of nonrandomised studies in meta-analyses. 2013; http://www.ohri.ca/programs/clinical_epidemiology/oxford.asp. Accessed October 1, 2016.

Copyright 2024 Precision AQ, LLC
